# Distributions of Irritative Zones Are Related to Individual Alterations of Resting-State Networks in Focal Epilepsy

**DOI:** 10.1371/journal.pone.0134352

**Published:** 2015-07-30

**Authors:** Yinchen Song, Basavaraju G. Sanganahalli, Fahmeed Hyder, Wei-Chiang Lin, Jorge J. Riera

**Affiliations:** 1 Department of Biomedical Engineering, Florida International University, Miami, Florida, United States of America; 2 Magnetic Resonance Research Center, Yale University, New Haven, Connecticut, United States of America; Universiteit Gent, BELGIUM

## Abstract

Alterations in the connectivity patterns of the fMRI-based resting-state networks (RSNs) have been reported in several types of epilepsies. Evidence pointed out these alterations might be associated with the genesis and propagation of interictal epileptiform discharges (IEDs). IEDs also evoke blood-oxygen-level dependent (BOLD) responses, which have been used to delineate irritative zones during preoperative work-up. Therefore, one may expect a relationship between the topology of the IED-evoked BOLD response network and the altered spatial patterns of the RSNs. In this study, we used EEG recordings and fMRI data obtained simultaneously from a chronic model of focal epilepsy in Wistar rats to verify our hypothesis. We found that IED-evoked BOLD response networks comprise both cortical and subcortical structures with a rat-dependent topology. In all rats, IEDs evoke both activation and deactivation types of BOLD responses. Using a Granger causality method, we found that in many cases areas with BOLD deactivation have directed influences on areas with activation (p<0.05). We were able to predict topological properties (i.e., focal/diffused, unilateral/bilateral) of the IED-evoked BOLD response network by performing hierarchical clustering analysis on major spatial features of the RSNs. All these results suggest that IEDs and disruptions in the RSNs found previously in humans may be different manifestations of the same transient events, probably reflecting altered consciousness. In our opinion, the shutdown of specific nodes of the default mode network may cause uncontrollable excitability in other functionally connected brain areas. We conclude that IED-evoked BOLD responses (i.e., activation and deactivation) and alterations of RSNs are intrinsically related, and speculate that an understanding of their interplay is necessary to discriminate focal epileptogenesis and network propagation phenomena across different brain modules via hub-based connectivity.

## Introduction

Functional magnetic resonance imaging (fMRI) has been used in a large number of studies to gain insights into the functional abnormalities of resting-state networks (RSNs) underlying various types of human epilepsy [1–11]. Decreased connectivity in brain networks, including the medial temporal lobe and the default-mode network (DMN), has been associated with the occurrence of interictal epileptiform discharges (IEDs) [1–8]. It has also been suggested that alterations in RSNs could facilitate the propagation of IEDs in patients with temporal lobe epilepsy [3]. Research has also shown that focal epilepsy might have a widespread detrimental effect through alterations in the integration and segregation of the whole brain interictal network organization [9]. However, the connection between the RSN topological alterations and the evolution of epileptogenesis has recently been challenged, since evidence shows that abnormal brain network organization might not be sufficient to induce epilepsy in those relatives of patients with idiopathic generalized epilepsy who share the same types of alterations in brain network [12]. Therefore, the relationships between IEDs and alterations in RSNs remain to be investigated further. It has been recently demonstrated that rats, a very useful preclinical model for epilepsy, also exhibits DMNs [13], providing a useful framework to explore this issue with experimental strategies less suitable for humans. Regrettably, we did not find any study about RSN in rat preclinical models of epilepsy.

Simultaneous electroencephalography (EEG) and fMRI constitutes the ideal technique to explore possible correspondence between IEDs and alterations in RSNs. It has been also extensively applied to understand hemodynamic and metabolic changes caused by neuronal activities in the epileptic brain, both for focal and generalized forms [14–20]. In these previous studies, confined increases in blood-oxygen-level-dependent (BOLD) signals evoked by IEDs, henceforth denoted as IED-evoked BOLD activation, are used to effectively localize irritative zones in patients with refractive focal epilepsy [21]. Although BOLD signal networks underlying seizure-type of activity have been extensively studied in rat preclinical models of different types of epilepsies [22–29], IED-evoked BOLD responses have received little attention. Human IED-evoked BOLD responses are often diffused, distantly distributed [19,30,31] and sometimes comprise brain areas with IED-evoked BOLD deactivations [14,17,18,30]. The mechanisms underlying both the spatial distribution of the IED-evoked BOLD response network and the emergence of such deactivations remain largely unknown. Gotman suggested that IED-evoked BOLD responses normally do not resemble a few isolated irritative areas, but extended networks with multiple locations [32], which includes the seizure initiating area of clinical interest. This point of view has been further supported by Fahoum et al., who pointed out that different epileptic syndromes would result in unique and widespread BOLD response networks with respect to the focal IEDs [30]. A crucial question yet to be addressed is whether or not these IED-evoked BOLD response networks are related to the alterations of RSNs mentioned above, for focal epilepsy in particular.

To objectively investigate and answer this question, we used a hierarchical clustering method to classify the type of epileptic network (i.e. focal/diffused and unilateral/bilateral) based on relevant features associated with the IEDs and the intrinsic RSN, extracted using EEG-fMRI data from a rat preclinical model of focal cortical dysplasia (FCD) with chronic seizures. First, we applied advanced mathematical tools to classify IEDs into different sub-types based on their temporal profiles, which is similar to the method utilized in the pilot study of classification of EEG abnormalities in humans with partial epilepsy [33]. IED sub-types with significant BOLD responses were considered genuine events. Each genuine IED sub-type might be related to a certain cortical or subcortical network inside the brain with either BOLD activation or deactivation. Second, we employed both seed-based regional pairwise correlation coefficient (RPCC) maps [34] and independent component analysis (ICA) [35] to obtain the spatial features associated with RSNs. The temporal features of genuine IEDs and the spatial features of RSNs were applied in the hierarchical clustering method to identify certain patterns in these features not directly perceivable from the raw data. To verify whether the spatial profile of the IED-evoked BOLD response networks were related to the alterations in the RSNs, the resulting patterns from the hierarchical clustering analysis were compared with the spatial distributions of IED-evoked BOLD responses. Note that no information about the IED-evoked BOLD response network was included in the clustering analysis. Finally, we used the Granger causality analysis for non-stationary signals to determine the directed influences, if any, between activated and deactivated regions of the IED-evoked BOLD response network for each rat. The rationale of using animal models instead of human subjects in this study will be explained in detail in the Discussion section.

## Materials and Methods

The animal study protocols were approved by the Institutional Animal Care and Use Committee (IACUC) at Florida International University (Approval Number: 13–065) and Yale University IACUC protocols (Approval Number: 11194) and carried out in full compliance with federal, state and local regulations and laws. All procedures were performed with anesthetized and sedated rats to minimize discomfort.

### Animal preparations

The “double-hit” FCD animal model was created with young adult male Wistar rats (~130 g, 4 weeks old), received from Charles River Laboratories, following the procedures described by Colciaghi et al. [36]. First, pregnant Wistar rats were treated with two doses of methylazoxymethanol acetate (MAM) (15 mg/kg maternal body weight, in sterile saline) intraperitoneally 12 hours apart on Embryonic Day 15 (E15) at Charles River Laboratories International, Inc. Twenty-five of their male offspring were delivered to Yale University. On Post-natal Day 28 (P28), they received one dose of N-methylscopolamine (0.5 mg/kg) intraperitoneally thirty minutes ahead of the intraperitoneal injection of pilocarpine (300 mg/kg). Henceforth, we will refer to this animal model as the MAM-PILO preclinical model of FCD. The status epilepticus onsets were determined by two investigators (YS and JJR) using the Racine scale [36]. Phenobarbital (20 mg/kg) treatments were delivered 90 minutes after the onset of status epilepticus to reduce mortality. Rats that had status epilepticus were continually monitored for a week to confirm they developed chronic seizures.

**Fig 1:**
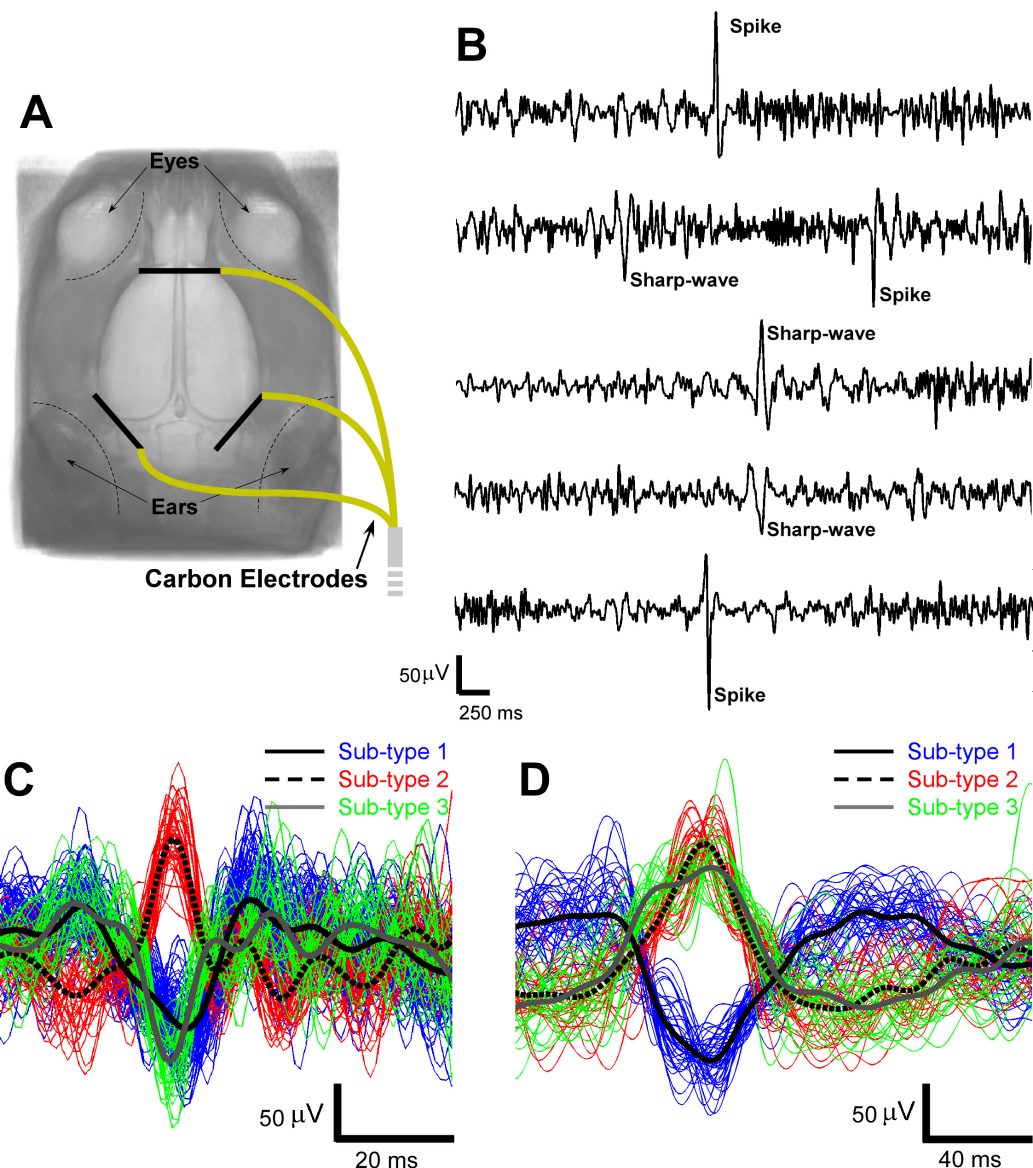
EEG acquisition and classification. (A) The locations of the three carbon electrodes implanted subcutaneously on top of the skull for EEG acquisition. (B) Representative EEG signals with artifacts removed and spike and sharp-wave marked. Color-coded sub-types of spikes (C) and sharp-waves (D). In both subplots, the black lines in different styles represent the averaged temporal profiles for each sub-type. EEG = electroencephalography.

Prior to the EEG-fMRI acquisition, anesthesia was induced using 5% isoflurane and later maintained at 1.5–2%. Three carbon-filament electrodes (1 mm in diameter, 8 mm in length, World precision Instruments) were placed subcutaneously on top of the rat skull (as illustrated in Fig 1A) and secured with cyanoacrylate adhesive (Leica Microsystem). After this minor surgical procedure, the anesthesia was switched to intravenous infusion of dexmedetomidine hydrochloride (~0.26 ml/hour, Dexdomitor, Orion Pharma). To that end, one tail vein was cannulated (PE10 polyethylene tubing, Instech Laboratories, Inc). The body temperature of the rats was monitored with a rectal probe and kept constant at 37°C using a water circulating heating pad (BAT-12 Microprobe Thermometer, Physitemp Instruments Inc.).

### EEG-fMRI acquisition

At the time of EEG-fMRI acquisition, the rats were about 8 weeks old and weighed around 250 g. During MRI recordings, each rat was fixed in a specifically designed plastic holder with its head positioned at the center. EEG signals were acquired using a Grass Data Recording System (Model 79D, Grass Instruments), low-pass filtered at 100 Hz, digitized and recorded (sampling rate, 2000 Hz) using PowerLab 8/35 data acquisition device and LabChart software (ADInstruments). All of these systems were located outside the MRI shielding room. MRIs were obtained on a modified 9.4T system with Varian (Agilent Technologies) spectrometer using a custom-built surface coil for transmitting and receiving radio frequency pulses at Yale University. We used single-shot gradient echo planar imaging (GE–EPI) for simultaneous EEG-fMRI recordings. GE–EPI data were acquired with the following parameters: repetition time (TR) = 2000 ms, echo time (TE) = 17 ms; field of view (FOV) = 25.6×25.6 mm, 64×64 matrix size; in-plane resolution = 390×390 μm; and slice thickness = 1000 μm. All GE–EPI experiments were acquired with 10 slices. The 10 slices were acquired over 2000 ms, followed by a 2-second pause before the next image onset. Time between the onsets of two consecutive image acquisitions was therefore 4 seconds. We acquired 450 images per experimental run, resulting in a total imaging time of 1800 seconds for a single experimental run. Sixteen dummy scans occurred before the receiver was turned on. Dummy scans were used to ensure that the proton spin system was in a steady state before data was collected. T2-weighted anatomical images for each animal were acquired with 10 interlaced slices in the coronal plane using fast spin echo multi slice (fSEMS), with TR = 4000 ms, TE = 9 ms, effective TE = 27 ms, FOV = 25.6×25.6 mm, and slice thickness = 1000 μm, without gap.

### EEG processing

Gradient artifacts from the MRI scanner were removed offline from the EEG recordings with the assistance of average artifact subtraction methods [37,38] and multiple band-stop filters. IEDs morphological patterns, including spikes (20–70 ms in duration, 15–50 Hz in frequency) and sharp-waves (70–200 ms in duration, 5–15 Hz in frequency), were detected automatically using the same method described by Pedreira et al. for polymorphic electrophysiological activity [33]. The distinctive temporal features of spikes and sharp-waves were extracted using the wavelet transform. To select the optimal features used for IED classification, the Anderson-Darling test and the kernel density estimation method were employed. Spikes and sharp-waves were subsequently classified into multiple sub-types using the mean shift clustering method without a prior knowledge of the number of sub-types. Afterward, the onset times of each spike or sharp-wave sub-type were convolved with the canonical hemodynamic response function (HRF) [39] to create the regressors for the general linear model (GLM) in fMRI analysis.

### MRI processing

Statistical Parametric Mapping (SPM8, http://www.fil.ion.ucl.ac.uk/spm) software package was used to analyze the T2-weighted images and GE-EPI recordings. The pre-processing of the GE-EPI images consisted of slice timing correction, realignment and spatial smoothing (Gaussian kernel, full width at half maximum = 0.6, 0.6, 1.5 mm). A MATLAB program developed in-house was used to remove artifacts of the T2-weighted images. Afterward, the T2-weighted images were reoriented with regard to the anterior commissure location and then co-registered with the averaged brain template for Wistar rats using predefined landmarks [40]. The same transformation for each rat was accordingly performed on the GE-EPI images. Changes in cortical structures have been associated with FCD lesions in this preclinical model [36]. However, a systematic evaluation using MRI data has not yet been performed. Therefore, a morphological analysis based on whole-brain T2-weighted images was performed in this study. Evaluating the existence of any consistent structural change in those cortical areas belonging to the RSN was of particular interest. The theoretical framework used in this study to perform the morphological analysis, which is based on the thin-plate splines non-linear registration method, is described in Valdés-Hernández et al. [40].

### IED-evoked BOLD response network

#### Topological organization

GE-EPI images were thereafter analyzed as an event-related design in SPM8 with all identified sub-types of spikes and sharp-waves as events (duration as zero). In each rat, one-tailed t-tests were performed to test for regional IED-evoked BOLD response, either positive or negative, with a rat brain mask applied and a threshold set at p<0.05 (family wise error, FWE, correction for multiple comparisons). Effects observed at a lower level of significance (p<0.001 or p<0.005, uncorrected) with a minimum cluster size (either 4 or 8 voxels) were also reported here. HRFs were extracted from all cortical regions with significant activations or deactivations in BOLD signals via FMRISTAT [41]. In FMRISTAT, the time course of the HRF is calculated by dividing each epoch into multiple blocks and estimating the correspondent effects of each block with autoregressive models, which does not require any assumption about the HRF shape. We statistically compared (Student t-test) the estimated HRFs at their peaks to show they were significantly different from zero (p<0.05). Identified brain regions with positive or negative IED-evoked BOLD response were illustrated in 3-D or 2-D rendering (positive: red; negative: blue) on the averaged brain template using MRIcroGL (http://www.mccauslandcenter.sc.edu/mricrogl by Chris Rorden), and separated into cortical and sub-cortical regions using the Wistar rat brain atlas [40] in SPM8. Cortical regions were further classified into different functional groups, such as motor region, sensory region, cingulate region, retrosplenial region and others in parietal and temporal regions. Statistical analysis was performed in Minitab to determine the distribution tendency of these IED-evoked positive and negative BOLD responses within above-mentioned cortical regions.

### Directed influence based on Granger causality

A data-driven Granger causality method [42] for non-stationary signals was employed to understand the information flow (i.e., effective connection) among IED-evoked brain regions. The MATLAB toolbox is available from a link provided by Luo et al. [42]. This novel method allows us to estimate the Granger causality from experimental datasets that comprise time-varying signals resulting from spontaneous brain activity oscillations. The method has been useful to find hidden hubs underlying causal relationships (e.g. the precuneus serves as a hub for information transfer in the brain) when classic Granger causality analysis has failed. Although it has been demonstrated that classic Granger causality is capable of identifying the direction of seizure propagation [25,43], there exist well-known evidences demonstrating significant oscillatory activity associated with IEDs as well [3,4,19,33]. Also, as a result of the spontaneous, irregular and unpredictable nature of IEDs, we believe the directed influences among IED-evoked brain regions could vary over time. We investigated and compared the directed influences among IED-evoked cortical and subcortical regions in terms of Granger causality. The time series applied in this method were the low-pass filtered (< 0.1 Hz) BOLD signals obtained from all the brain regions related to a certain sub-type of IEDs. We designated the BOLD signals from the IED-evoked BOLD activation area as **P** (positive), and those from the IED-evoked BOLD deactivation area as **N** (negative). There are seven possible directions of the influence between two different IED-evoked brain regions, denoted by **P**→**N**, **N**→**P**, **P**→**P**, **N**→**N**, **P**↔**N**, **P**↔**P**, and **N**↔**N**, where the arrows show the significant direction of influence (p<0.05, at least). An understanding of such propagations between brain regions with IED-evoked BOLD activation and deactivation is helpful to gain insight to their underlying mechanisms. We did not follow David et al.’s suggestion of de-convolving the BOLD signal [25] with the estimated region-specific HRF, for reasons discussed later.

### Resting-state network

#### RPCC and ICA maps

To study the RSN (i.e., functional connectivity) in each rat, we used two different well-known approaches: a) the regional pairwise correlation coefficient (RPCC) maps [34] and b) the independent component analysis (ICA) [35]. To create the RPCC maps, the BOLD signal for each voxel was first band-pass filtered from 0.01 Hz to 0.08 Hz. Then, a single BOLD time series was created for each cortical region of the segmented brain atlas for Wistar rats [40] by instantaneously averaging the filtered BOLD signal within the region. The RPCC maps were calculated by lag-zero cross-correlating the BOLD time series from all cortical regions. The RPCC maps directly show patterns of connectivity between resting-state BOLD signals from anatomically connected and/or unconnected cortical regions. However, RPCC maps fail to account for the functional variability (i.e., IEDs or normal resting-state activities) within certain cortical regions. To avoid the bias resulting from using region-dependent analysis, individual resting-state connectivity analysis was voxel-wisely performed on the basis of ICA (group ICA of fMRI toolbox, GIFT) [35], which follows a temporal concatenation approach.

RPCC and ICA maps are very complex and show significant inter-subject variability, making it very difficult to quantitatively compare them among rats and, therefore, to raise any useful conclusion. Consequently, these maps have to be reduced to a set of features useful to detect hidden patterns. The numbers of paired-regions with positive (RPCC_+_, >0.5) or negative (RPCC_−_, <-0.5) cross-correlations in the RPCC maps were introduced to detect asymmetries in these maps with respect to the cerebral hemispheres. We proposed this feature based on previous findings about the bilateral character of the DMN in rats [13], and its alterations in some neurological disorders, like stroke [44]. We introduced an index of similarity (S_RPCC_) among the RPCC maps for different rats by using principle component analysis (PCA), where S_RPCC_ = weighted-sum of the first-two PCA scores. This index of similarity will allow us to discriminate robust RSN components from those vulnerable to change because of epileptiform activity. Finally, ICA components were classified into two classes (bilateral and unilateral), based on the previously mentioned bilaterality of RSN in normal rats. The total number of ICA components (ICA_TOT_) and the number of components in each class (ICA_BI_−bilateral and ICA_UNI_−unilateral) were estimated.

#### The hierarchical clustering analysis

From a naked-eye inspection, it was difficult to perceive any correlation between these RSN features and the topologies of the IED-evoked BOLD response network. To objectively explore the existence of such correlates, we performed a hierarchical clustering analysis, a well-known method in data mining, on features RPCC_+_, RPCC_−_, S_RPCC_, ICA_BI_, ICA_UNI_ and ICA_TOT_. In addition, we included in this analysis the temporal features of genuine IEDs. Genuine IEDs are those evoking significant BOLD responses satisfying both the GLM analysis and HRF peak-based t-test criteria. For each rat, the total number of genuine IED events (N_IED_), the number of genuine IED sub-types (N_TYPE_) and the average frequency (f_AVE_) per sub-type during the entire recording were estimated. These temporal features do not contain any spatial information of the IED-evoked BOLD response network, but might reflect indirectly the abnormalities in the organization of RSNs. Epileptic network classes obtained from the hierarchical clustering analysis were subsequently compared with those determined by the spatial distribution of irritative zones.

## Results

A total of seven rats developed chronic seizures. One of these rats was excluded due to poor GE-EPI quality. Hence, six rats that had IED activities during the GE-EPI acquisitions were included in this study.

### IED detection and classification

After removal of the gradient artifacts from the recorded EEG signals, spikes and sharp-waves were detected separately (Fig 1B). The total number of spikes and sharp-waves detected from each rat during the 1800-second time window varied, as shown in Table 1. The detected IEDs were classified into multiple sub-types as shown in Fig 1C and 1D.

**Table 1:** Frequency of Interictal Epileptiform Discharges (IEDs).

Rat	Spike	Sharp-wave
**1**	142	158
**2**	116	231
**3**	38	79
**4**	7	52
**5**	15	92
**6**	331	284

### Topological organization of irritative zone network

**Fig 2:**
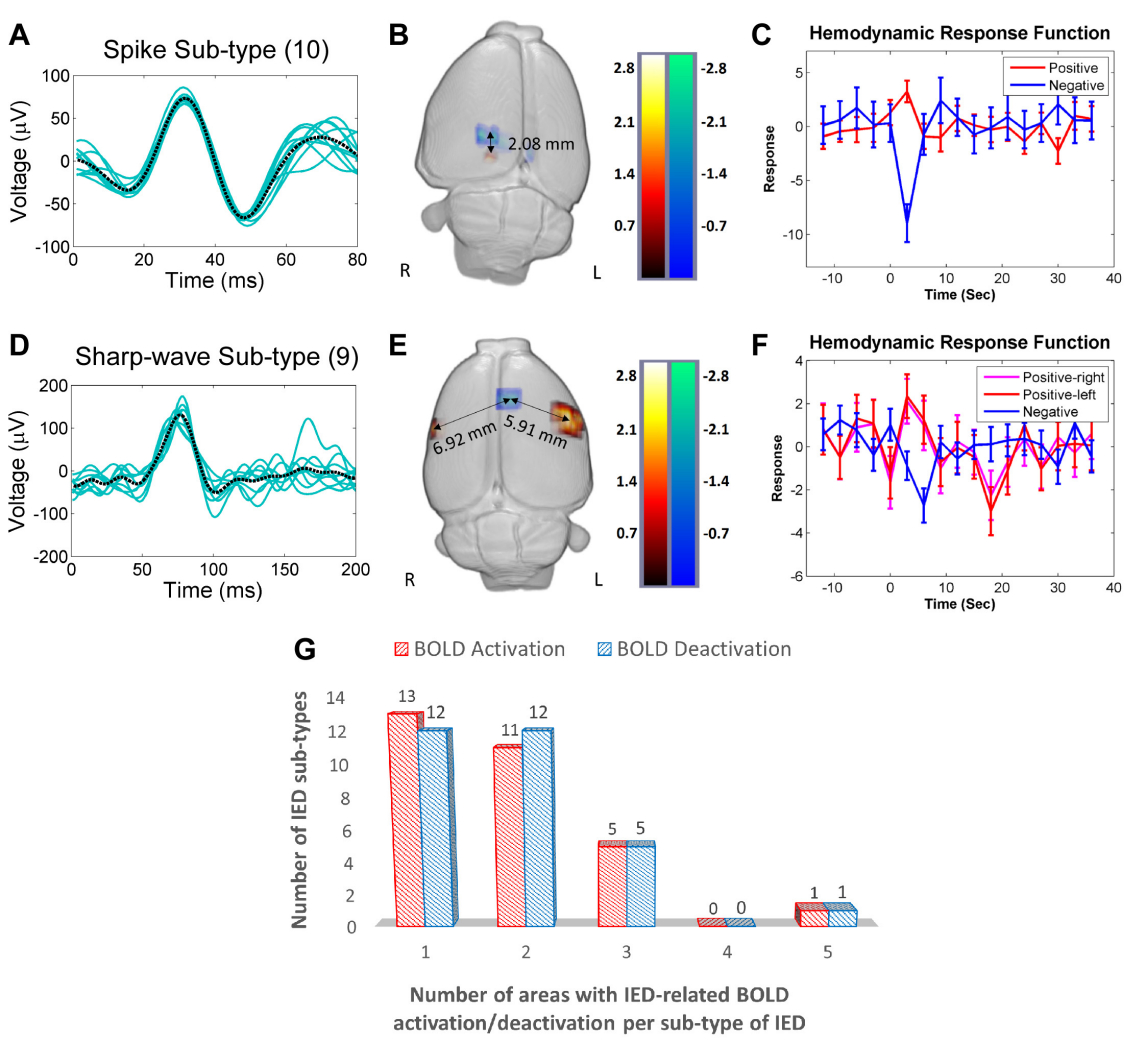
IED-evoked BOLD activations and deactivations. (A) A representative collection of a particular spike sub-type (10 events, Rat 6). The black dashed line represents the average of all events. (B) 3-D display of brain regions with either positive (red) or negative (blue) BOLD responses evoked by the spike sub-type shown in (A). In this case, the distance between the BOLD responses is 2.08 mm. (C) Hemodynamic response functions (HRFs) extracted from the regions with a positive and a negative BOLD responses shown in (B). (D) A representative collection of a particular sharp-wave sub-type (9 events, Rat 1). The black dashed line represents the average of all events. (E) 3-D display of the regions showing either a positive (red) or a negative BOLD response evoked by the spike sub-type shown in (D). The distances between the region with a negative BOLD response and those two with a positive BOLD response are 6.92 mm (right hemisphere) and 5.91 mm (left hemisphere). (F) HRFs extracted from the regions with either a positive or a negative BOLD response shown in (E). (G) Numbers of the positive (red) and the negative (blue) BOLD regions versus the IED sub-type. For example, among all the studied rats, there are thirteen sub-types of IEDs that induce BOLD activation in a single brain area. R = right; L = left; BOLD = blood-oxygen-level dependent; IED = interictal epileptiform discharge.

For certain sub-types of IEDs, the corresponding activations and/or deactivations in the BOLD response formed clusters that were localized in both subcortical and cortical areas. Fig 2 shows two different examples of topological distributions of irritative zones. Upper and lower panels shows the waveforms (Fig 2A and 2D), evoked BOLD responses (Fig 2B and 2E) and HRFs (Fig 2C and 2F) for a particular spike and a sharp-wave sub-type, respectively. In both cases, positive (red) and negative (blue) BOLD responses were observed. The distances between the voxel with the maximum value for the positive BOLD response and the one with the minimum value for the negative BOLD response were calculated for each IED sub-type (Fig 2B and 2E). The maxima or minima of the estimated HRFs were found significantly different from zero (p<0.05). Genuine IEDs for each rat (Table 2) were therefore determined by two criteria: first the GLM analysis and then this HRF peak-based t-test.

**Table 2:** Frequency of Classified Genuine Interictal Epileptiform Discharges (IEDs).

Rat (Total number of IEDs)	Spike		Sharp-wave	
	Sub-type	Number	Sub-type	Number
**1 (108)**	1	84	1	11
	2	6	2	7
**2 (175)**	1	70	1	85
			2	12
			3	8
**3 (53)**			1	24
			2	21
			3	8
**4 (16)**			1	16
**5 (73)**	1	3	1	64
			2	4
			3	2
**6 (237)**	1	87	1	46
	2	47	2	8
	3	15	3	3
	4	8		
	5	7		
	6	7		
	7	3		
	8	3		
	9	3		

**Fig 3:**
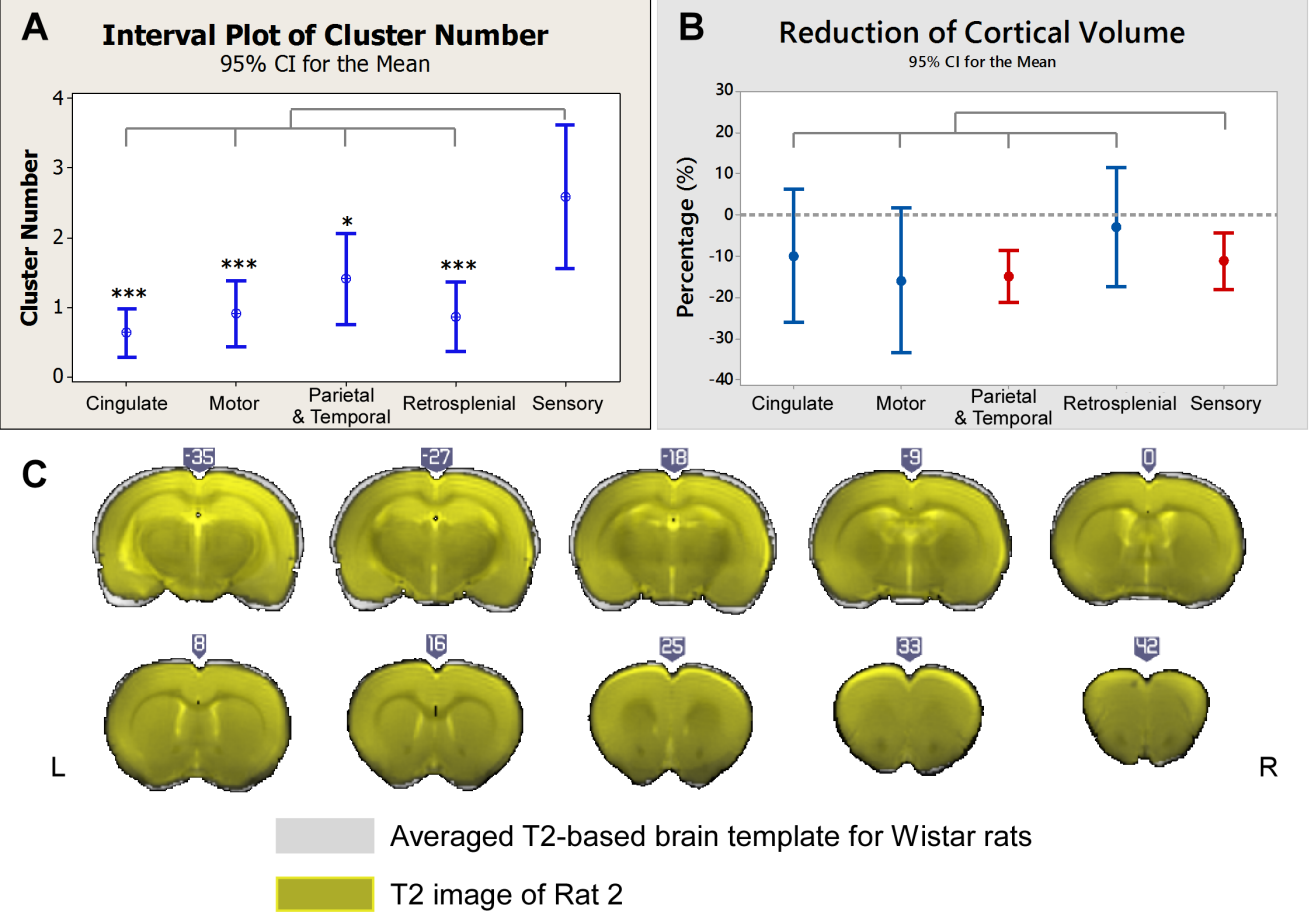
Distributions of irritative zones and morphological changes. (A) Comparisons of the numbers of IED-evoked BOLD response clusters for five cortical regions. The numbers of clusters in the sensory areas were significantly larger than those in other cortical areas. (B) Comparisons of the volume reduction in the same five brain regions as depicted in (A). The dash line indicates zero-change. The volume reductions in sensory and other cortices in the parietal and the temporal regions were significant (p < 0.05). No significant differences were found among cortical regions. (C) Coronal T2-weighted images of Rat 2 (in yellow) overlapped with the averaged brain templates (in grey) after registration (illustrated in MRIcroGL). Clear reduction in the cortical thickness was found in the anterior commissure. All compared to sensory cortex. *: p<0.05. **: p<0.01. ***: p<0.001. No asterisk means no significant difference. IED = interictal epileptiform discharge; BOLD = blood-oxygen-level dependent.

The majority of IED-evoked BOLD responses (80%) were found within one or two areas in the brain, as shown in Fig 2G, which reflects the focal nature of IED initiation in this type of focal epilepsy. IED-evoked BOLD response clusters were predominantly localized within the sensory cortices (Fig 3A), including somatosensory, auditory and visual areas. The morphological analysis indicates a significant volume reduction in the sensory cortical areas, as well as in the parietal and temporal regions (Fig 3B). Fig 3C shows the coronal brain slices of Rat 2 (in dark grey) superposed with the averaged template (in grey), as an example, from which we could see Rat 2’s neocortex was thinner than the averaged template–caudal to the anterior commissure.

In this section, we illustrated the IED-evoked BOLD response network obtained from some rats. In the last section of **Results**, we will provide the topological networks for all rats involved in this study.

### Directed influence within the irritative zone network

**Fig 4:**
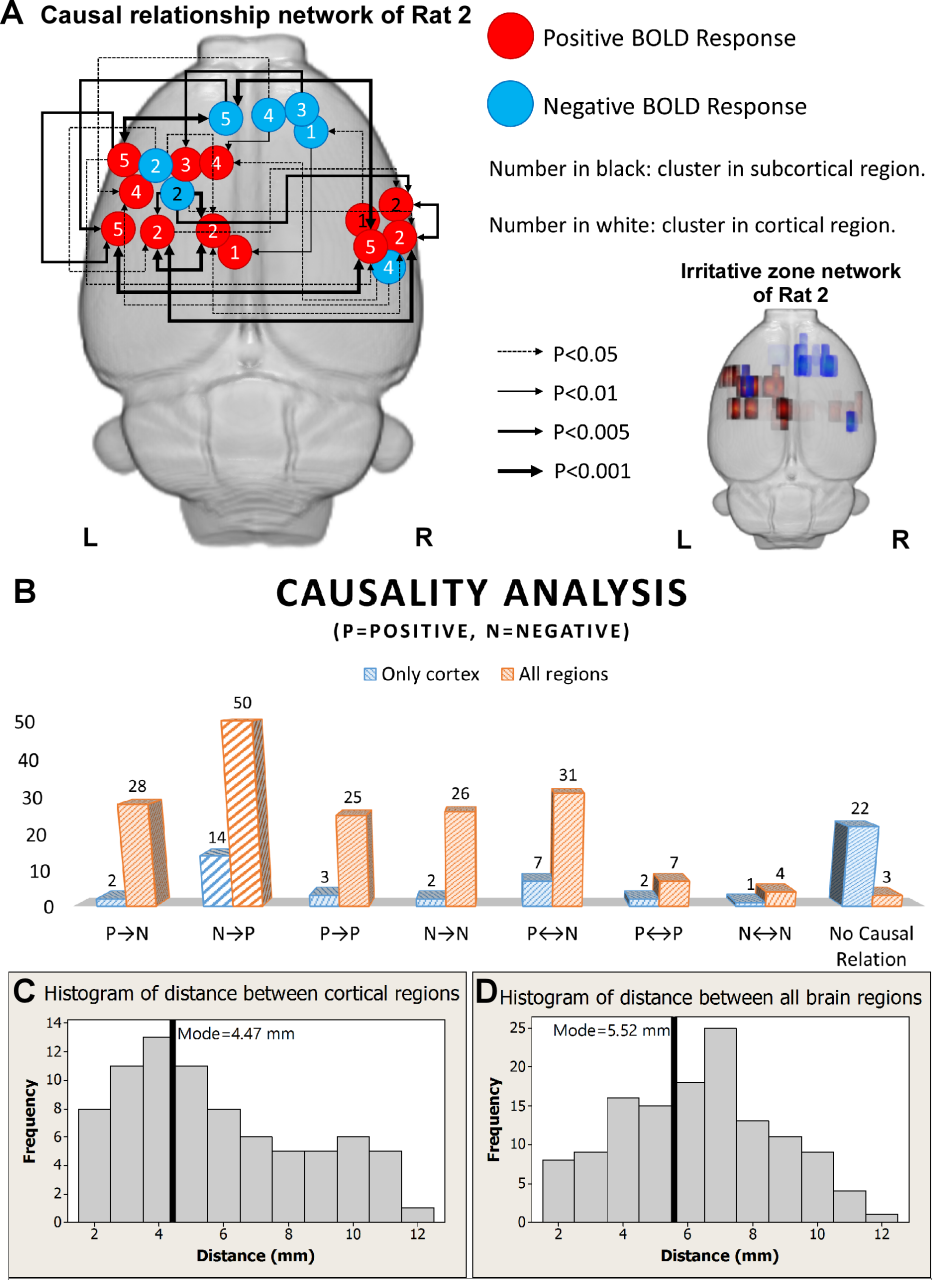
Granger causality analysis. (A) Illustration of the causal relationship among BOLD response clusters evoked by all identified sub-types of genuine IEDs (n = 5) for Rat 2 (left). The actual locations and extensions for all BOLD response clusters are shown on the right (activations—red and deactivations—blue). The predominant relationship shown here was that BOLD deactivations have a directed influence on activations. (B) Summary of the Granger causality analysis for all six rats. The blue bars were from the results of the analysis including only clusters in the cortex. Most of these clusters showed no causal relationships (n = 22). The orange bars were from the analysis performed including clusters from both the cortical and subcortical regions. Both analyses depicted that predominantly areas with BOLD deactivations have directed influences on areas with activations. (C) Histogram of the distance between cortical areas with activation and deactivation. The mode of the distribution is 4.47 mm, marked by black solid line. (D) Histogram of the distance between activation and deactivation but, in this case, including areas from the entire brain, i.e. cortical and sub-cortical regions. The mode of the distribution is 5.52 mm, marked by the black solid line. R = right; L = left; BOLD = blood-oxygen-level dependent; IED = interictal epileptiform discharge.

We used results from Rat 2 to illustrate the network properties and the causal relationships for both BOLD activations and deactivations triggered by each IED sub-type (Fig 4). Note that for this particular rat the irritative zones form a middle-anterior network (Fig 4A, right). The causal relationships between regions for the same IED sub-type were illustrated using arrows with various thicknesses, which indicate the strengths of the network connections (Fig 4A, left). As an example, we discuss the particular case of IED sub-type No. 4 (number inside circles). This IED sub-type was generated in the right cingulate cortex with a negative BOLD effect, which propagated (directed influence) to the contralateral motor cortex (p<0.01) and somatosensory cortex for forelimb (S1FL) (p<0.05) where it created a positive BOLD response. Meanwhile, the same IED evoked a negative BOLD response in the right parietal cortex (posterior area, rostral part PtPR), which propagated to cause a BOLD activation in the same motor cortex (p<0.05) and S1FL (p<0.05). Note that cingulate cortex and PtPR are parts of the default mode network in rats [13]. A comprehensive inspection of all these network connectivity maps for Rat 2 suggested that in several cases neuronal activity evoking BOLD deactivations propagates to other regions where it evokes a BOLD activation. This result was confirmed by a quantitative analysis of all possible cluster pairs in cortical regions within the same IED sub-type (**P**→**N**, **N**→**P**, **P**→**P**, **N**→**N**, **P**↔**N**, **P**↔**P**, and **N**↔**N**). Letters **P** and **N** stand for positive and negative BOLD responses, respectively. Therefore, symbol **P**→**N** indicates a directed influence from an area with a positive BOLD response to an area with a negative BOLD response.

The IED-evoked BOLD response networks and activation/deactivation causal relationships were examined for all the remaining rats. We found a large variability in the spatial organization of these networks among the rats, which indicated that the re-organization of the epileptic network is subject-dependent in focal epilepsy. However, the analysis confirmed that cortical areas with BOLD deactivations mostly have directed influence on cortical areas with BOLD activation, which might be reflecting activity propagation within the cortical network (n = 14, Fig 4B, blue bars). However, we noticed that some cortical regions with either activated or deactivated BOLD responses, were not effectively connected to any other cortical areas (n = 22, Fig 4B, blue bars). When subcortical areas were also included in this analysis, the number of total disconnected brain regions was reduced (n = 3). This result indicates that effective connections between cortical and subcortical regions exist in this preclinical model, regardless of the network variability among the studied rats. Nevertheless, directed dynamical interactions happen majorly from regions with BOLD deactivations to regions with activations, reflecting a principal direction for the propagation of IEDs (n = 50, Fig 4B, orange). Therefore, areas with BOLD activations could sometimes just result from the network propagation phenomenon.

Predominantly, the distance between activated and deactivated cortical areas, according to their BOLD responses, followed a bimodal distribution (Fig 4C) with about 32% of the pairs closer than 3 mm. This might also suggest a role of local vascular reorganization around these close cluster pairs producing blood stealing and leakage phenomena in the neocortex. In contrast, when all brain areas (cortical and subcortical) were included, the distances between activation-deactivation clusters were approximately normally distributed with mean around 5.52 mm (Fig 4D), which is approximately the half of the brain size for these rats. Therefore, these epileptic networks would cover the entire brain of the rat in this case.

### Abnormalities in the resting-state connectivity maps

**Fig 5:**
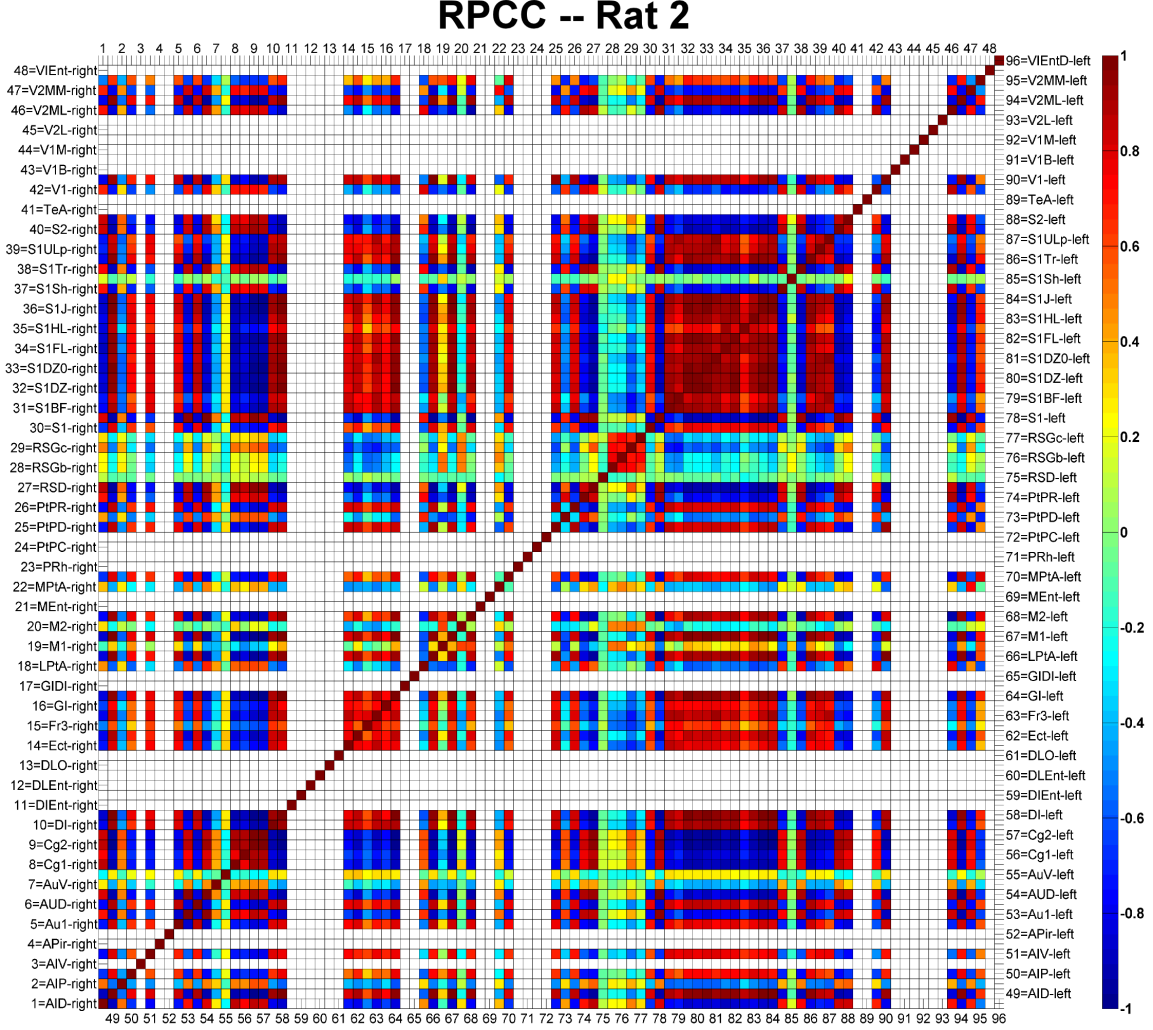
Example of the regional pairwise correlation coefficient (RPCC) map of the averaged resting-state time series in the cortical regions of Rat 2. Numbers 1–48 represent cortical regions on the right hemisphere and numbers 49–96 represent cortical regions on the left hemisphere. The abbreviations of the cortical regions are defined by Paxions et al. [45]. Areas not covered by the echo planar imaging acquisitions are left blank. The color-bar represents the RPCC value; dark red indicates strong correlation (r>0.8) and deep blue strong anti-correlation (r<-0.8).

The RPCC map of Rat 2 is shown in Fig 5 as an example. Each cortical area was assigned with a number ranging from 1 to 96, of which the first half were on the right hemisphere and the rest were on the left hemisphere. The names of all cortical areas were written using the abbreviations provided by Paxions et al. [45]. The diagonal line of the matrix is, by definition, equal to 1, since it represents the correlation of a signal with itself. Cortical areas not covered by the GE-EPI images were left blank in the RPCC matrix. The cingulate cortices were highly correlated (r>0.8, p<0.05) with the auditory and visual cortices on the right hemisphere, while they were strongly anti-correlated (r<-0.8, p<0.05) with those on the left hemisphere. In addition, the cingulate cortices (Cg1 and Cg2) were strongly anti-correlated (r<-0.8, p<0.05) with most of other cortical areas in both hemispheres (i.e., motor, most of somatosensory and some other cortical areas). Meanwhile, highly anti-correlations (r<-0.8, p<0.05) were found between hemispheres for the auditory and the visual cortices. The left and right somatosensory cortices were strongly correlated (r>0.8, p<0.05). On the other hand, the retrosplenial granular cortex (c region, RSGc) was moderately correlated (0.3<r<0.4, p<0.05) with the cingulate cortices and the right motor cortices, and moderately anti-correlated (-0.4<r<-0.3, p<0.05) with the somatosensory cortices and the left motor cortices. Similar RPCC patterns were observed in three (Rat 1, Rat 2 and Rat 4) out of the six rats. The important features associated with the RPCC matrices of all rats (S1, S2, S3, S4 and S5 Figs) are summarized in Table 3.

**Table 3:** Summary of all Analysis.

Rat	IED			RPCC			ICA-based RSN		
	N_IED_	N_TYPE_	f_AVE_	RPCC_+_	RPCC____	S_RPCC_	ICA_TOT_	ICA_BI_	ICA_UNI_
**1**	108	4	27.00	18	2	0.406	27	12	15
**2**	175	4	43.75	16	9	0.357	25	16	9
**3**	53	3	17.67	0	0	0.083	6	3	3
**4**	16	1	16.00	10	5	0.293	14	11	3
**5**	73	4	18.25	1	1	0.088	8	4	4
**6**	237	12	19.75	0	0	-0.006	7	0	0

IED: interictal epileptiform discharge

N_IED_: the total number of genuine IED events

N_TYPE_: the number of genuine IED sub-types

f_AVE_: the average frequency per sub-type of IED

RPCC: regional pairwise correlation coefficient

RPCC_+_: the number of paired-regions with positive (>0.5) cross-correlation in RPCC maps

RPCC_: the number of paired-regions with negative (<-0.5) cross-correlation in RPCC maps

S_RPCC_: index of similarity among the RPCC maps for different rats (S_RPCC_ = weighted-sum of the fist-two scores of principal component analysis)

ICA: independent component analysis

RSN: resting-state network

ICA_TOT_: the total number of ICA component

ICA_BI_: the number of IC components with bilateral distribution

ICA_UNI_: the number of IC components with unilateral distribution.

**Fig 6:**
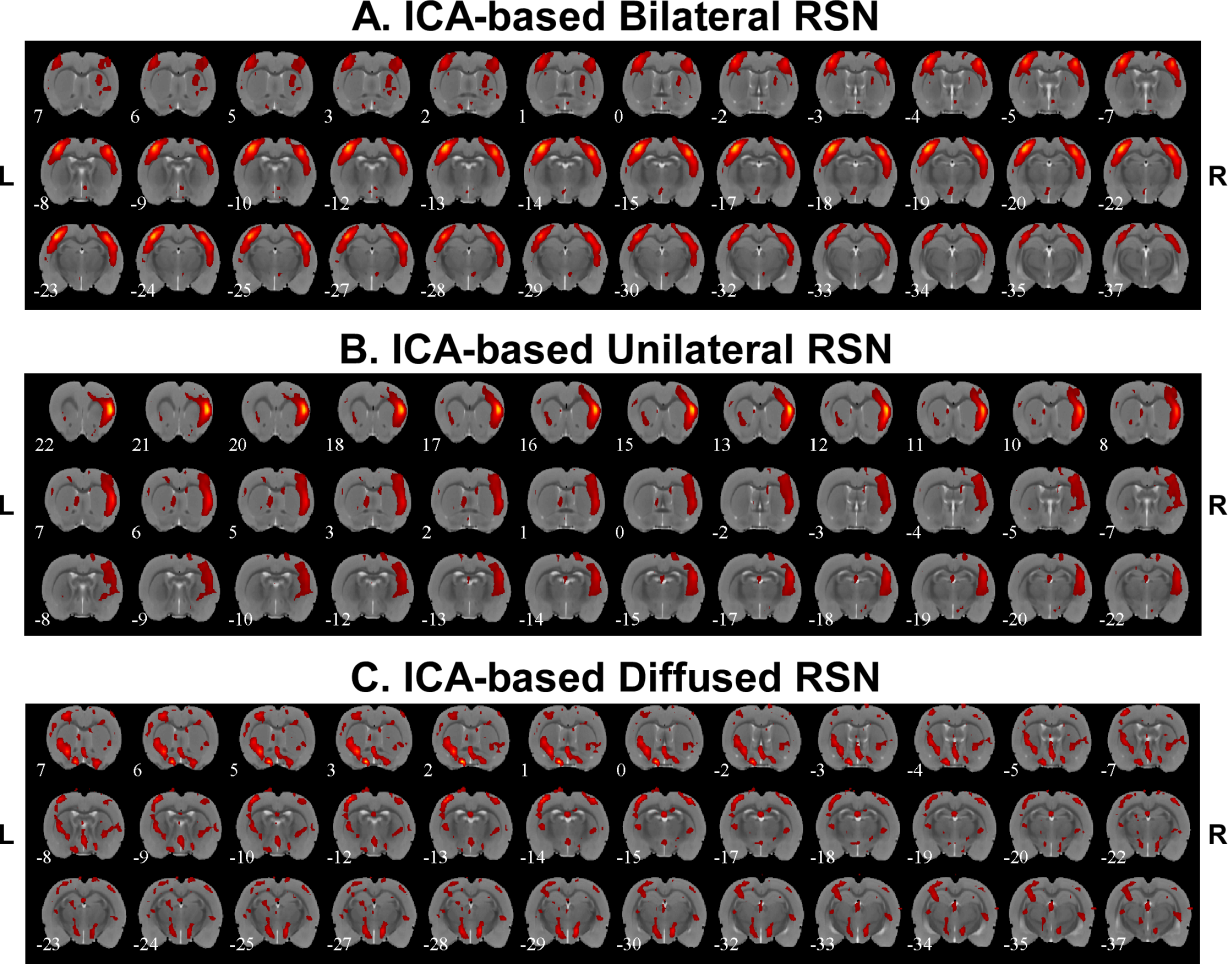
Independent component analysis (ICA)-based resting state networks. A) Example of bilateral connectivity (Rat 4). B) Example of unilateral connectivity (Rat 2). C) Example of diffused connectivity (Rat 6). R = right; L = left.

Spatial features associated with RSNs were also extracted by applying ICA to the low-pass filtered BOLD signals. Fig 6 demonstrated three different connectivity networks we identified from these six rats, which reveal the existence of bilateral connectivity (Fig 6A), unilateral connectivity (Fig 6B) and diffused connectivity (Fig 6C). Bilateral RSN connectivity was found among somatosensory cortices for all the rats with the exception of Rat 6 that seemed to have mainly diffused subcortical RSN components. Bilateral connectivity was also seen in Rat 3 and Rat 5 for the auditory cortices. The number of bilateral and unilateral connectivity networks for each rat is summarized in Table 3.

### Hierarchical resting-state connectivity clusters

**Fig 7:**
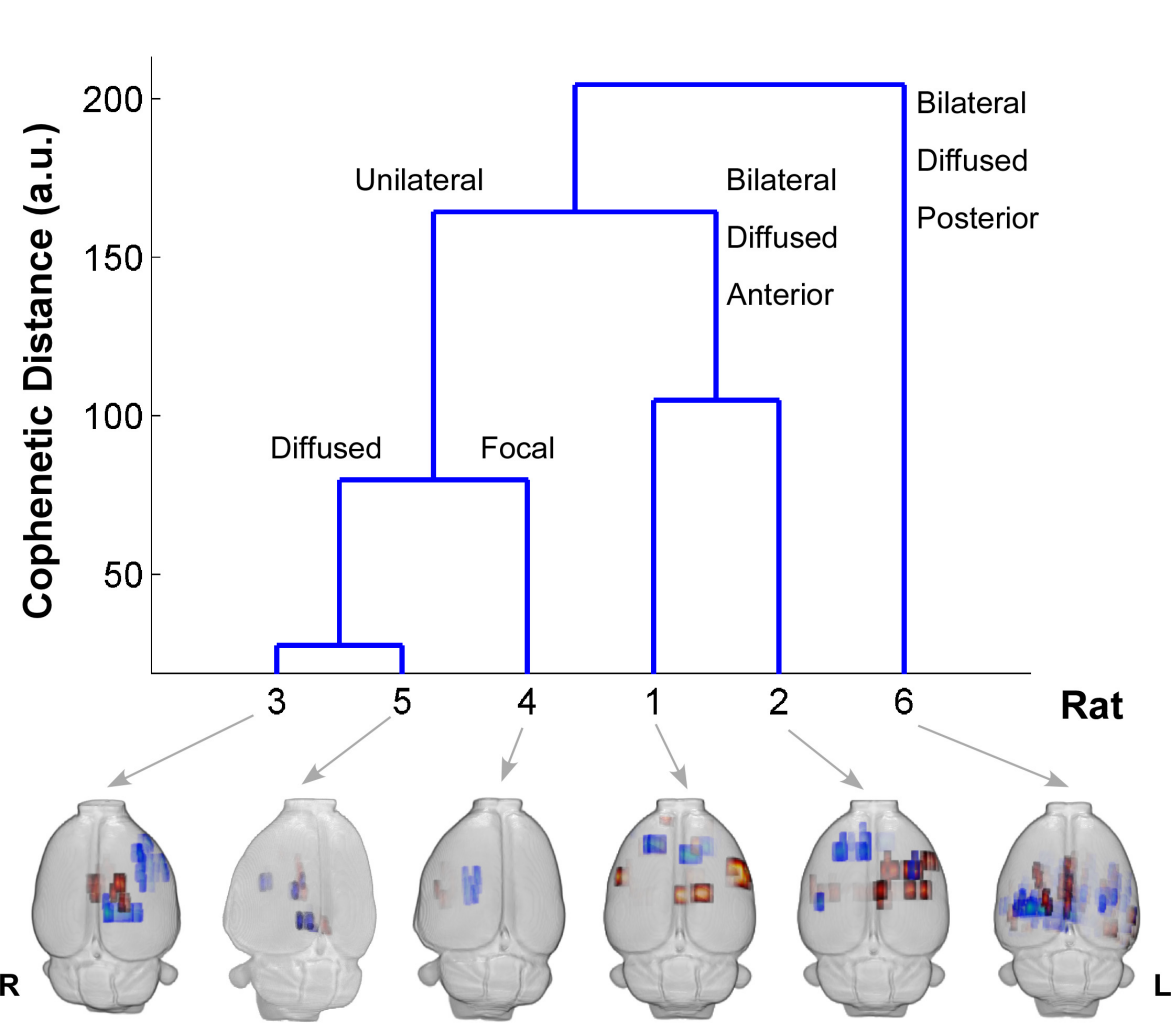
Hierarchical Clustering. The blue cluster tree is obtained using the data in Table 3 as the input for hierarchical clustering. The height of the cluster tree is the cophenetic distance between two different classes in an arbitrary unit. The horizontal axis is the rat number. All the labels on the top of the blue lines represent descriptions of the common feature of the irritative zone distribution within each class. The 3-D BOLD response maps of the distribution of irritative zones obtained from these six rats are displayed below the cluster tree. R = right; L = left; BOLD = blood-oxygen-level dependent.

The first three columns of Table 3 show frequency of genuine IEDs events for each studied rat. The middle three columns are related to important spatial features from the seed-based RPCC analysis. The last three columns show important spatial features related to the bilateral or unilateral pattern of the ICA-based RSNs. We used all features in Table 3 to perform a hierarchical clustering analysis. The cluster tree in Fig 7 shows that these six rats could be classified into different groups depending on the selection of the threshold for the cophenetic distance. We compared this clustering result with the actual distribution of irritative zones (maps on the bottom), and verified that the classes, which were obtained from the hierarchical clustering based mostly on spatial properties of RSNs, were related to the unique spatial distribution patterns of the irritative zones. It should be noted that no information about the spatial distribution of the irritative zones was included in this clustering analysis, which demonstrates that important altered spatial features of the RSNs in focal epilepsy are related to the organization of the irritative zones.

## Discussion

### Are the IED sub-types actual paroxysmal events?

In this study, we successfully detected the IEDs at two different frequency bands with auto-thresholding and classified them into multiple sub-types with mean shift clustering methods. The first two questions that came to our mind while reviewing the IEDs obtained with our detection and classification methods were: 1) Are they actually reflecting paroxysmal events? 2) Are they different from those observed in patients with focal epilepsy? To answer these questions we investigated the following three critical aspects: the methodology used to identify the IEDs, as well as the frequency and the temporal profiles of the IEDs.

Our design for automatic detection was based on a combination of several filtering techniques and a thresholding principle. We know that spikes and sharp-waves have characteristic temporal windows, 20–70 ms and 70–200 ms, respectively. Therefore, we could separate them by designing appropriate filters with the assistance of filter design and analysis tool (fdatool) in MATLAB Signal Processing Toolbox. A common characteristic of the used filters is that they cut out low frequency fluctuations, which is a required pre-process to employ a thresholding method. Regarding the classification of these two types of IEDs into sub-types by means of the mean shift clustering, we have found similar methodologies being utilized in human studies (ICA [46] and superparamagnetic clustering [33,47]). These studies demonstrated the feasibility of going beyond typical visual inspections by using sophisticated mathematical tools to detect small variations in the temporal profiles of each type of IEDs. Such variations could be due to differences in location and activation sequences of the brain sources underlying these IEDs. This was particularly needed in our study, because of the limitation of our experimental design due to the use of only three electrodes. With such a fine classification, we believe IEDs from different brain regions were segregated during the BOLD signal analysis with individual regressors. As a matter of fact, the method for detection and classification applied in this study on a preclinical model is similar to those used in the human studies referred above.

In terms of the IED frequency and temporal profiles, there are compelling results from previous studies on humans and rats that support our findings in this MAM-PILO preclinical model. First of all, it has been shown that the frequency of IEDs ranges from ~1/min to 47/min for humans [33,46,48,49] and around 3/min for rats [50]. In our study, the combined frequency of spikes and sharp-waves were between 0.5/min and 9/min, which are in good agreement with these previous studies. Traditionally, clinicians identify IEDs based on the standard waveforms obtained from the bipolar EEG recordings. By selecting the bipolar montage with the largest IED responses, clinicians have dealt with possible variability introduced by changes in the location inside the brain. However, other studies have demonstrated that such large variability is more notable when using EEG data from multiple channels with respect to a common reference [51]. In our study, we used three electrodes strategically placed (see Fig 1A) to detect IEDs originated from any part of the rat brain, as a result of which IEDs from different brain regions must have different waveforms. In addition, by comparing side-by-side IEDs detected in this study with those reported in previous studies [50–52], we were able to confirm that they resemble typical waveforms of paroxysmal events in focal epilepsy.

### BOLD activation/deactivation: distribution and relationship

We found that each specific genuine IED sub-type generally caused BOLD responses in one or two focal brain area(s). However, it might be sometimes diffused, which was the case for one of the six rats in this study, and has been reported in human focal epilepsy studies as well [46,48,49,51,52]. First of all, our study provides the first fMRI-based evidence showing that epileptogenesis in such a well-defined model of FCD occurs not only in subcortical, but also in cortical regions. As a consequence of the scattered nature of the cortical malformations in these MAM rats and the low-spatial resolution of electrophysiological techniques, it has been difficult in the past to demonstrate the cortical origin of IEDs [36].

As we mentioned previously, the mechanisms underlying BOLD deactivation, its relationship with activation in the epileptic networks, and its value in localizing irritative zones or even seizure onset zones, still remains largely unknown to date. We were able to untangle these issues here by utilizing the Granger causality method to understand the relationships between IED-evoked BOLD activation and deactivation. We found that area with IED-evoked BOLD deactivation has directed influence on area with activation, even though they were not in the immediate vicinity of each other, reflecting long-range connections within the epileptic network. However, we found that cortical areas with BOLD deactivations were, in many cases, in close proximity to regions with a BOLD activation. We speculate here that such close deactivation/activation pairs might reflect cortical vascular reorganization caused by sustained hyper-excitability. We have recently found such vascular reorganization in the epileptic cortices of humans ([52–55], preliminary data from our lab).

It has been suggested by David et al. [25] that miss-estimation in the causal relationships among brain regions could be introduced by not de-convolving the BOLD signal with the particular HRFs. Performing the deconvolution can be an important tool, but it is crucially dependent upon assumptions and characteristics of the data [56]. In the particular case of having multiple scalp electrodes, one can solve the inverse problem to obtain estimates of the particular neuronal activity at each brain area, which will conserve the actual event timings. An elegant way to deal with this problem is by introducing a Dynamic Causal Model (DCM) [57] for each brain region involved and used it to reproduce the macroscopic EEG and fMRI data simultaneously. In this case, one can estimate parameters of the model using EEG and fMRI data, as well as compare different models using the data evidence. There has been a study using DCM to study causality of seizure propagation based on EEG-fMRI data [58]. Implicitly, such a method will be able to conserve the actual timings for neuronal activity in different brain region. Thus, estimations of the causal relationships will be more truthful if the assumed models are correct. However, the use of a deconvolution could adversely introduce biases in the results obtained from limited number of scalp electrodes. In our study, we employed only three electrodes. Hence, we cannot solve the EEG inverse problem using data from three electrodes, making it impossible to precisely determine the temporal activation sequence in all involved brain areas. We believe performing a HRF deconvolution using such limited EEG information could introduce undesirable errors. In David et al.’s study [25], considerable variations in the shape of the HRF reported for different brain regions could be the reason underlying miss-estimations of the actual causal relationships. For example, the HRF for the somatosensory barrel cortex of genetic absent epilepsy rats from Strasbourg (GAERS) was significantly slower than those for the other brain regions of interest. We did not observe any substantial variation in the shapes of HRFs obtained from all irritative zones (Fig 2C and 2F). We did find significant variations in their amplitude, probably reflecting changes in the neurovascular coupling strength, as it has been demonstrated by using intracranial recordings (unpublished data from our lab). Therefore, we performed the Granger causality analysis on the non-deconvolved BOLD signals. By using a theoretical framework and computer simulations, a recent paper demonstrated that Granger causality analysis is invariant to HRF convolution [59].

### Abnormal RSN in focal epilepsy

We identified different patterns (i.e., bilateral, unilateral and diffused) in the RSNs of the studied rats, both with RPCC maps and ICA-based methods. An understanding of what would be an abnormal pattern in the rat RSN is crucial for our analyses. Jonckers et al. performed a series of experiments on Sprague-Dawley rats under the same medetomidine analgesics and demonstrated that normal RSN usually consists of the following bilateral sub-networks [60]: 1) motor, 2) somatosensory, 3) auditory, 4) RSG, 5) hippocampus, 6) striatum, 7) cingulate, 8) visual, and 9) inferior colliculus. The most important appearance of the rat RSN is the bilateral distribution of each component, compared to the unilateral distribution for mice [60]. Rat brains have a DMN similar to humans, which is related to a variety of cognitive functions and includes hippocampus, orbital cortex, cingulate cortex, auditory/temporal association cortex, retrosplenial cortex, et cetera [13]. Decreased connectivity within the DMN has been found in a lot of neurological and psychiatric disorders/diseases [61], such as Alzheimer’s, autism, spectrum disorders, attention deficit hyperactivity disorder (ADHD), and also in patients with epilepsy. Our results indicated the existence of alterations in various sub-networks of the rat DMN across the studied rats, of which the “bilateral feature” was compromised. A similar preclinical biomarker was also observed in rats with stroke [44]. In a particular case of epilepsy, alterations of RSNs might be a consequence of IED-evoked perturbations of executive and alertness/attention functions. A previous behavior study from our lab on rats with temporal lobe epilepsy has demonstrated deficits in decision-making were positively associated with the severity of epilepsy [62]. Thus, investigation of RSN of rats with focal epilepsy should be continued on a larger population and monitored multiple times at different ages in conjunction with behavior studies, as a result of which we might be able to gain new and important insights into the reorganization of the brain during the development of chronic seizures.

### Paroxysmal EEG events and RSN alterations: are they related?

**Fig 8:**
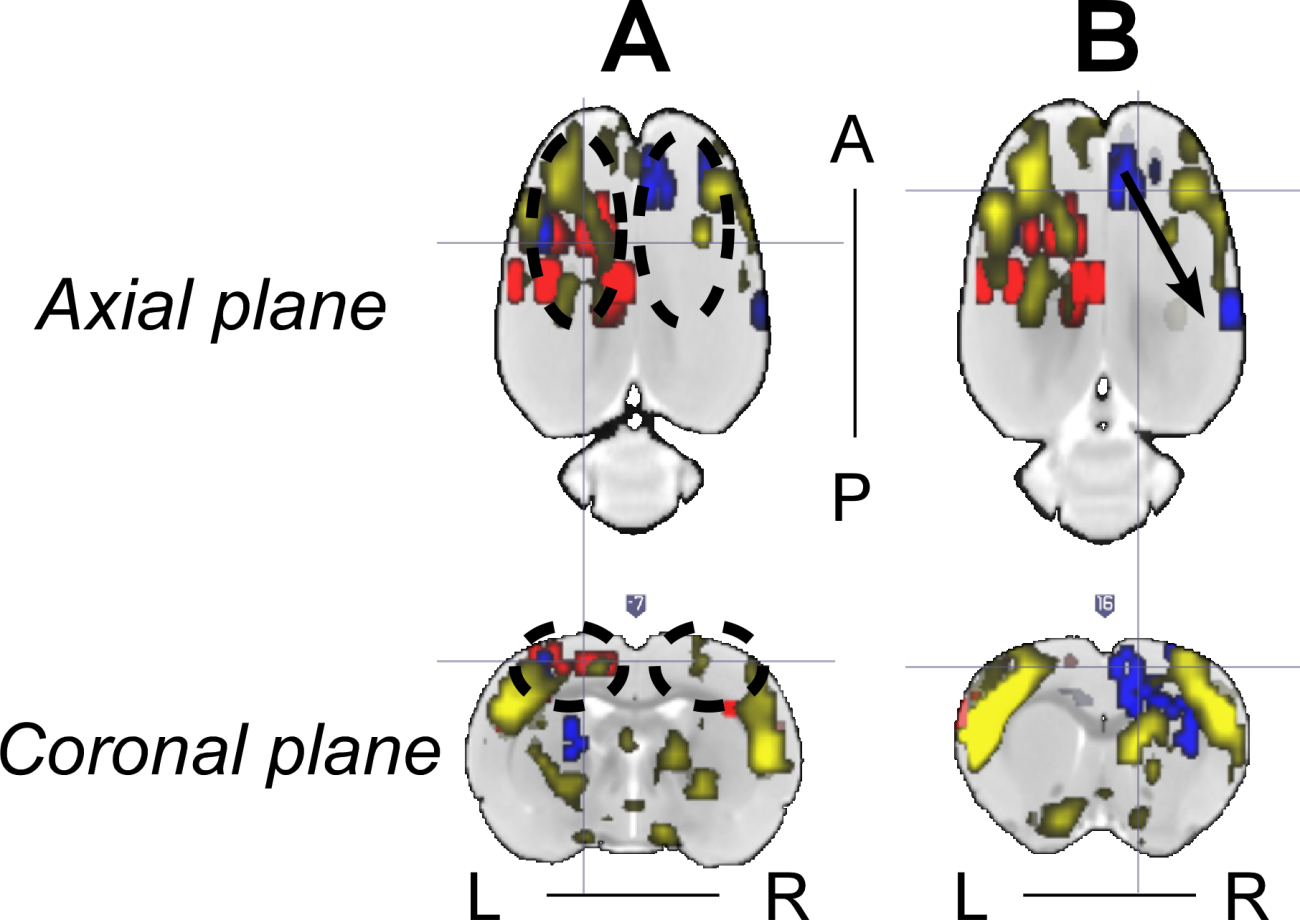
Representative relationship between the IED-evoked BOLD responses and RSN from Rat 2. (A) and (B) show two different axial and coronal slices with IED-evoked BOLD responses overlapped with RSN. RSN’s symmetry was disturbed in (A) due to the existence of two IED-evoked BOLD responses (black dash circles). Overlap between RSNs and IED-evoked BOLD responses exists preferentially on the left hemisphere. (B) Distant IED-evoked BOLD responses are connected by RSN (black arrow). IED = interictal epileptiform discharge; BOLD = blood-oxygen-level dependent; RSN = resting-state network; R = right; L = left; A = anterior; P = posterior.

We demonstrated that the frequency of genuine IED events and the alterations in the RSNs, as determined by the RPCC and ICA methods, correlate very well with the distribution of the irritative zones (Fig 7), although it appears that these features are rather rat-dependent. Therefore, all these features together might be uncovering the same intrinsic epileptic networks in the brain. To demonstrate the complexity of such a relationship, we extended the discussion of Figs 4 and 5 along with other important findings. The RSN of the same rat in Figs 4 and 5 shows asymmetries in some ICA components that seem to have a relationship with the IED-evoked BOLD response network. For example, part of the RSNs (yellow) was overlapped with the IED-evoked BOLD activation/deactivation (red/blue) on both hemispheres (Fig 8A and 8B). Apparently, some distant areas associated with the same sub-type of IED (Fig 4A) were linked by a sub-network of the RSN. Our findings indicate that the existing RSN could play a role in effectively facilitating the propagation of IED events from one area to a distant one, even if they do not belong to the same sub-network of RSN, or are on different hemispheres. As for their anatomical connections, it exceeds the scope of current study, which focuses on the relationship between IEDs and their disturbance in RSNs, and remains to be examined in future studies.

Our observation is supported by previous findings in human studies, which studied the IED-evoked disturbance in default-mode areas of patients with temporal lobe epilepsy (TLE) and suggested that alterations of the default-mode network could also facilitate IED propagation [3]. Compared to normal subjects, patients with mesial TLE, demonstrated disrupted causal connectivity between the epileptogenic zone and other brain regions [63]. It has also been found that the distribution of the network hubs was altered in TLE patients compared to control subjects [1]. More hubs were identified within the area associated with epileptogenesis. Focal epilepsy causes increases in local connectivity with concurrent decreases in global or long-distance connectivity [64,65], thus disturbing the RSN. The existence of increased hubs within the epileptogenic area could facilitate the propagation of IEDs. It could also increase the possibility to affect a remote area with the IED event through the remaining links in RSN (Fig 8B). The role played by network organization in the evolution of brain disorders constitutes a hot topic of recent studies [12,64–68]. Graph theory analysis has been employed to understand the reorganizational changes secondary to epilepsy inferred from various measures of neuronal activities, including fMRI and EEG [65,67,68]. It has been demonstrated that epileptic discharges could result from either abnormalities in the dynamics of brain regions or the connectivity structures between them [68]. However, evidence has been raised to question the role of abnormalities of brain networks in epileptogenesis related to idiopathic generalized epilepsy (IGE) [12], as mentioned in the Introduction. This is a very important finding with regard to the mechanistic understanding of IGE, though cautions should be given to imply its validity in focal epilepsy, which remains to be investigated.

**Fig 9:**
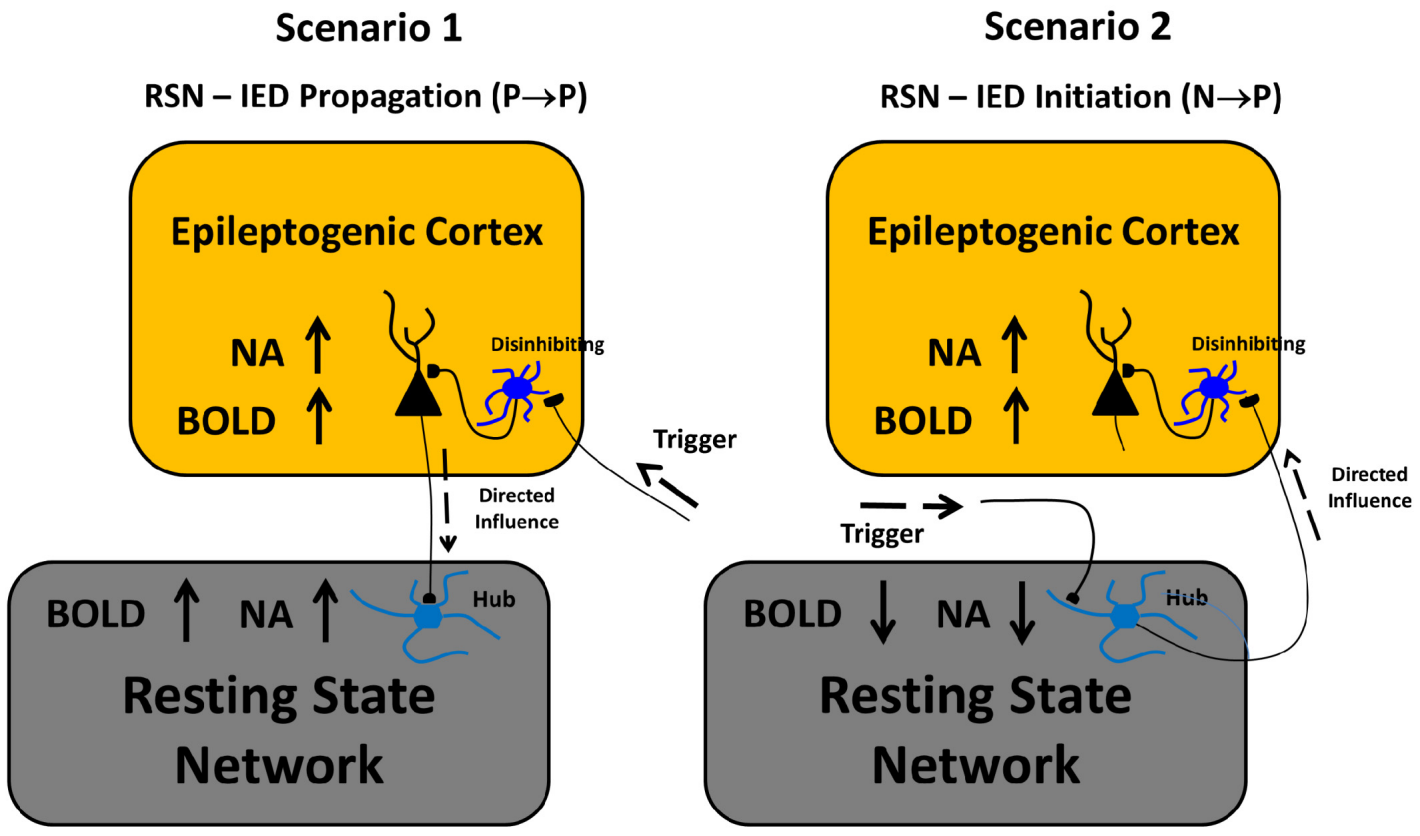
Scenarios. Two possible scenarios by means of which the resting-state network and the irritative zone network might be intrinsically related. A mechanistic explanation for the directed influences among elements from the irritative zone network are provided. NA = neuronal activity; BOLD = blood-oxygen-level dependent; IED = interictal epileptiform discharge; RSN = resting-state network.

Similar to the human brain, an organization of the connectome (i.e., small-world, modular and rich club) was recently demonstrated to exist in normal rats as well [69], which suggests the above mentioned conclusions for focal epilepsy patients might also be valid in rats with focal epilepsy. As demonstrated in the Granger causality analysis (Fig 4A), IEDs initiated at one particular area that acts like a functional hub have directed influences on activities evolved in multiple areas, either proximal or distal. In other words, IED activity propagation (Scenario 1, Fig 9) might take advantage of existing functional highways linking different modules through specific hubs. The alterations caused by IEDs, whether they are generated from one or multiple focal area(s), could have an effect on the entire network via the highway-based facilitating mechanisms. Moreover, the degree of disturbance of the RSN might depend on the relative locations of the irritative zones with respect to these highways. Therefore, we believe that alterations of RSNs are caused by IEDs, and meanwhile, they play a supporting role to facilitate the propagation of IEDs within certain sub-networks. In our opinion, the final distributions of the irritative zones, as shown in Fig 7 for different rats (i.e., focal/diffused, unilateral/bilateral), will be determined by the relative location of the epileptogenic cortex and the functional network hubs; hence affecting the RSN as well. Such a theory justifies our results from the hierarchical clustering analysis, providing a new analytical tool to evaluate the epileptic network distribution, the results of which is complementary to those obtained using simultaneous EEG-fMRI recordings. In this context, the use of BOLD deactivation to localize epileptogenic cortices, which has been questioned in the past [19], will depend on its relative location with respect to the RSN. A Granger causality analysis might help decipher the actual epileptic network topology. For regions inside the RSN, BOLD deactivations may result from inhibition of the RSN activity that might initiate hyperactivity in the form of IEDs in other brain regions (Scenario 2, Fig 9) according to our Granger causality analysis, with which a positive BOLD signal could be associated. Alternatively, some of these IED-evoked BOLD deactivations could have a pure vascular origin (e.g. blood stealing effect); therefore, for them **N**→**P** type of causal relationship will not be the case.

### Rationale of using the “double-hit” model of FCD

#### Inter-subject homogeneity

We decided to use an animal model instead of human subjects, because of the difficulties associated with establishing a homogeneous study population (i.e. identical seizure types and frequency, as well as similar etiologies). Animal models also offer the possibility to perform invasive histopathological, electrophysiological and behavioral studies precisely by the help of pharmacological manipulations and/or through disease modeling. Clearly, they have the potential to provide more insight and understanding of human focal epilepsy, as it has been demonstrated in other EEG-fMRI studies using different seizure models in rats [15,20,70]. Additionally, epileptic patients might undergo different neurological or psychiatric conditions (i.e., claustrophobia) during the recordings, which could cause some nuisance effects to the RSN and IED-evoked BOLD responses, while animals could be sedated and maintained under the same physiological conditions during the entire acquisition procedure. Therefore, animal models of focal epilepsy are of great importance to investigate the alterations of the RSNs and the interplay between BOLD activation and deactivation with regard to the irritative zone. Therefore, the inter-subject variability found in this study for the RSN patterns and the related irritative zone topologies have an intrinsic physiological origin, and were not caused by external factors (e.g. psychological states, misclassification of the epilepsy type).

#### Common pathophysiological features

Recently, Colciaghi et al. investigated the morphologic and molecular bases of the “double-hit” model of FCD in rats [36]. These authors demonstrated that epileptic activities could be detected through scalp EEG. We know that FCD is very commonly diagnosed, especially in pediatric patients [71–73]. Therefore, questions were raised regarding this preclinical model in particular, in terms of its proximity to the human FCD. To clear our doubts, we would first review typical features identified in human FCD cases and then check if they are manifested in this preclinical model as well. In humans, FCD could be classified into multiple types based on different histopathological markers. For example, the major feature of type I FCD is the cortical dyslamination. In addition to the cortical dyslamination, type II FCD includes the presence of dysmorphic neurons with neurofilament over-expression, increased parvalbumin-positive puncta and disoriented dendrites [55,74–77]. Similar hypertrophic/dysmorphic pyramidal neurons exist in this preclinical model [36]. GABAergic terminals enveloping the dysplastic neurons and NMDA receptor complex alterations observed in these neocortical abnormalities bear similarities with those observed in the preclinical model.

Another feature that could further separate type II FCD into two sub-categories (IIA and IIB) is the existence of balloon cells. If there are other types of lesions other than type I and II pathologies, it could be categorized into type III FCD. In this preclinical model, it has been demonstrated that the cortical layering abnormalities are severe [78], especially in more lateral and caudal cortical areas, after the prenatal treatment of MAM. Besides, the presence of abnormal pyramidal neuron with enlarged soma and nuclear size and apical dendrite thickness in the MAM-PILO-treated rat cortex was observed [36]. However, the existence of balloon cells has not been spotted yet, although the role of balloon cells in epileptogenesis has been questioned for some time [79]. Based on these findings, it seems this animal model of FCD resembles histopathological features of type IIA FCD, which shows cortical dyslamination and dysmorphic neurons, but without the presence of balloon cells.

For type II FCD, the presence of focal cortical thickening, which usually coexist with poorly defined transitions between gray and white matter and the hyperintensity of the subcortical white matter, has been used as a marker to localize the lesion [80]. In contrast, cortical thinning has been found in this preclinical model [36,78]. Accordingly, we found a significant volume reduction in the sensory cortices (i.e., somatosensory, auditory and visual cortex) and observed IED-evoked BOLD activation and deactivation more frequently in the sensory cortices, which is consistent with findings by Colciaghi et al. about the cortical atrophy in somatosensory cortical areas [36]. They claimed that progressive cortical, as well as hippocampal, atrophy of this “double-hit” model of FCD were positively related to seizure frequency and severity. It has been also reported that the decrease of cortical thickness is related to durations of seizure and network alterations in other human focal epilepsy studies [1,81], but not in FCD. It was suggested that the cortical thickening in patients with FCD is probably due to the convolution of gyri [82], which is one of the unique structural features of human brain that rodents do not possess. Our results from the morphological analysis pointed out that structural changes occur in the sensory cortex and most of the parietal cortical areas, which are part of the rat’s RSN. Further studies need to be conducted to evaluate whether these are intrinsic changes due to the abnormal cortical development or are caused by sustained epileptiform activity propagating along RSN during seizure perpetuation. The fact that cortical thinning was also observed in some temporal areas, which are not part of the RSN, indicates that they might be due to alterations of the normal process of cortical ontogenesis [36].

### Future considerations

Simultaneous EEG-fMRI and RSN analyses are both promising and complementary to help understand altered brain networks induced by sustained IEDs in patients with focal epilepsy. Pittau et al. proposed to combine BOLD response and EEG source analysis using dense electrode arrays to improve focus localization in patients with focal epilepsy [21], with which we completely agree and have done so with our patented mini EEG cap for rats, in particular on the same set of FCD rats [83]. As for further validation, the spatiotemporal profiles of hemodynamic and metabolic changes caused by epileptic activities in areas with IED-evoked BOLD responses were studied in vivo by means of laser Doppler flowmetry, intrinsic optical imaging and multi-electrode-array-based electrophysiology [84]. These authors suggested the use of region-/animal- dependent hemodynamic response function to obtain reliable estimators of the underlying epileptic networks. Of particular interest is the variation in the HRF peak-amplitude, which might reflect changes in the neurovascular coupling strength. Histology of the suspicious irritative zone should also be conducted to verify the existence of actual malformations in cortical structures with either BOLD activation or deactivation. Above all, to practice the pre-surgical evaluations of IED-evoked BOLD response and alterations of RSNs in a clinical realm is also very essential. These two analyses could be achieved from the same acquisition session, which is cost-effective. Therefore, we are now working with Miami Children’s Hospital and Florida Hospital Orlando to validate our results on pediatric and adult epileptic patients.

## Conclusions

In summary, this study demonstrated that alterations of RSNs were related to the topology of IEDs network activity. Such alterations manifested as reductions in connectivity of various sub-networks of the default-mode network. The fact that the epileptic network organization was properly classified via hierarchical clustering with genuine IED temporal features and RSN spatial alterations provides a promising analytical tool, even when concurrent EEG and fMRI recording is not available. In our study, each resultant group from such a classification method encompasses a unique spatial distribution of the irritative zones. We speculated that such correlation is a consequence of the linking between the irritative zones and the hubs of various modules in the RSNs.

The existence of such hub-based highways facilitates the propagation of IEDs within the brain. We anticipate that similar approaches based on resting-state fMRI and EEG-fMRI analysis can be used for other different types of focal epilepsy in a clinical realm to enhance the efficiency of source localization and to gain more insights into the human brain networks under pathological conditions. Furthermore, areas with IED-evoked BOLD deactivations in the IED-based epileptic network mostly had directed influence on areas with activations somewhere else. Connected brain areas with activation and deactivation in BOLD responses were either distant (Fig 2E), probably reflecting propagation through a hub-based network connectome, or in very close proximity (Fig 2B), perhaps resulting from local blood stealing/leakage phenomena. Our results endorse the use of fine IED classification methods proposed in the past [33] as a crucial step to localize all irritative zones in the epileptic network. This study is the first to demonstrate that epileptogenesis occurs also in the neocortex of the MAM-PILO rats, an up-to-date missing link between anatomical and functional aspects in such a relevant preclinical model of FCD. Cortical irritative zones in MAM-PILO rats were more frequently localized within sensory cortices, but more studies are required to explain why significant volume reduction is prone to occurring in these cortical areas.

## Supporting Information

S1 Fig Regional pairwise correlation coefficient (RPCC) matrix of the averaged resting-state time series in the cortical regions of Rat 1. Numbers 1–48 represent the cortical regions on the right hemisphere; 49–96 on the left hemisphere. The abbreviations of the cortical regions are defined by Paxions et al. [45]. Areas not covered by the echo planar imaging acquisitions are left blank. The color-bar represents the RPCC value; dark red indicates strong correlation (r>0.8) and deep blue strong anti-correlation (r<-0.8).
(TIF)

S2 Fig Regional pairwise correlation coefficient (RPCC) matrix of the averaged resting-state time series in the cortical regions of Rat 3. **Text same as in S** 1.
(TIF)

S3 Fig Regional pairwise correlation coefficient (RPCC) matrix of the averaged resting-state time series in the cortical regions of Rat 4. **Text same as in S** 1.
(TIF)

S4 Fig Regional pairwise correlation coefficient (RPCC) matrix of the averaged resting-state time series in the cortical regions of Rat 5. **Text same as in S** 1.
(TIF)

S5 Fig Regional pairwise correlation coefficient (RPCC) matrix of the averaged resting-state time series in the cortical regions of Rat 6. **Text same as in S** 1.
(TIF)
