# Supplementary figures and images for: Distributions of Irritative Zones Are Related to Individual Alterations of Resting-State Networks in Focal Epilepsy
Source: PLoS One. 2015 Jul 30;10(7):e0134352. doi: 10.1371/journal.pone.0134352 (PMC4520590; doi:10.1371/journal.pone.0134352)

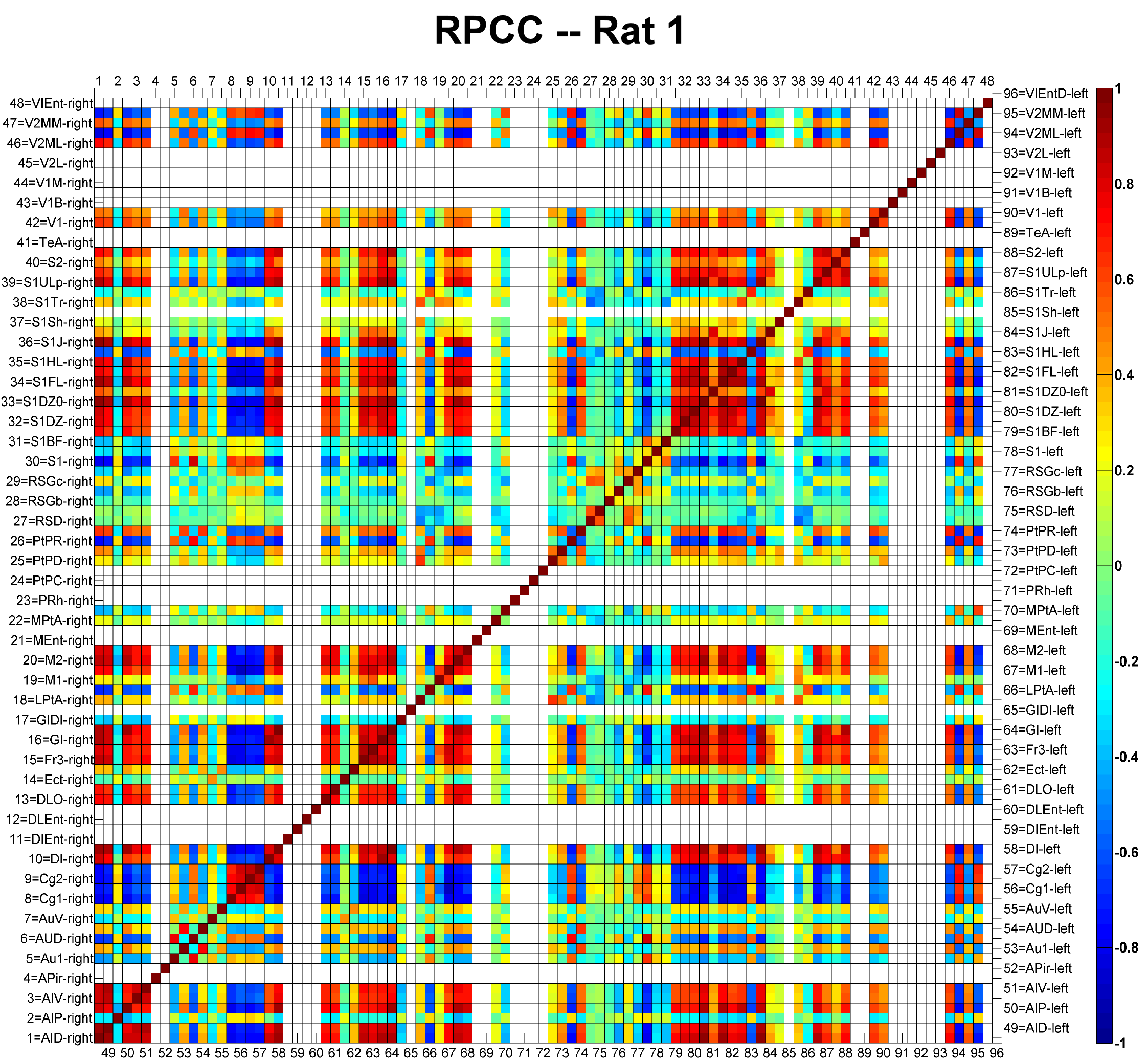

Supplement: S1 Fig — Numbers 1–48 represent the cortical regions on the right hemisphere; 49–96 on the left hemisphere. The abbreviations of the cortical regions are defined by Paxions et al. [45]. Areas not covered by the echo planar imaging acquisitions are left blank. The color-bar represents the RPCC value; dark red indicates strong correlation (r>0.8) and deep blue strong anti-correlation (r<-0.8). (TIF) [file pone.0134352.s001.tif]

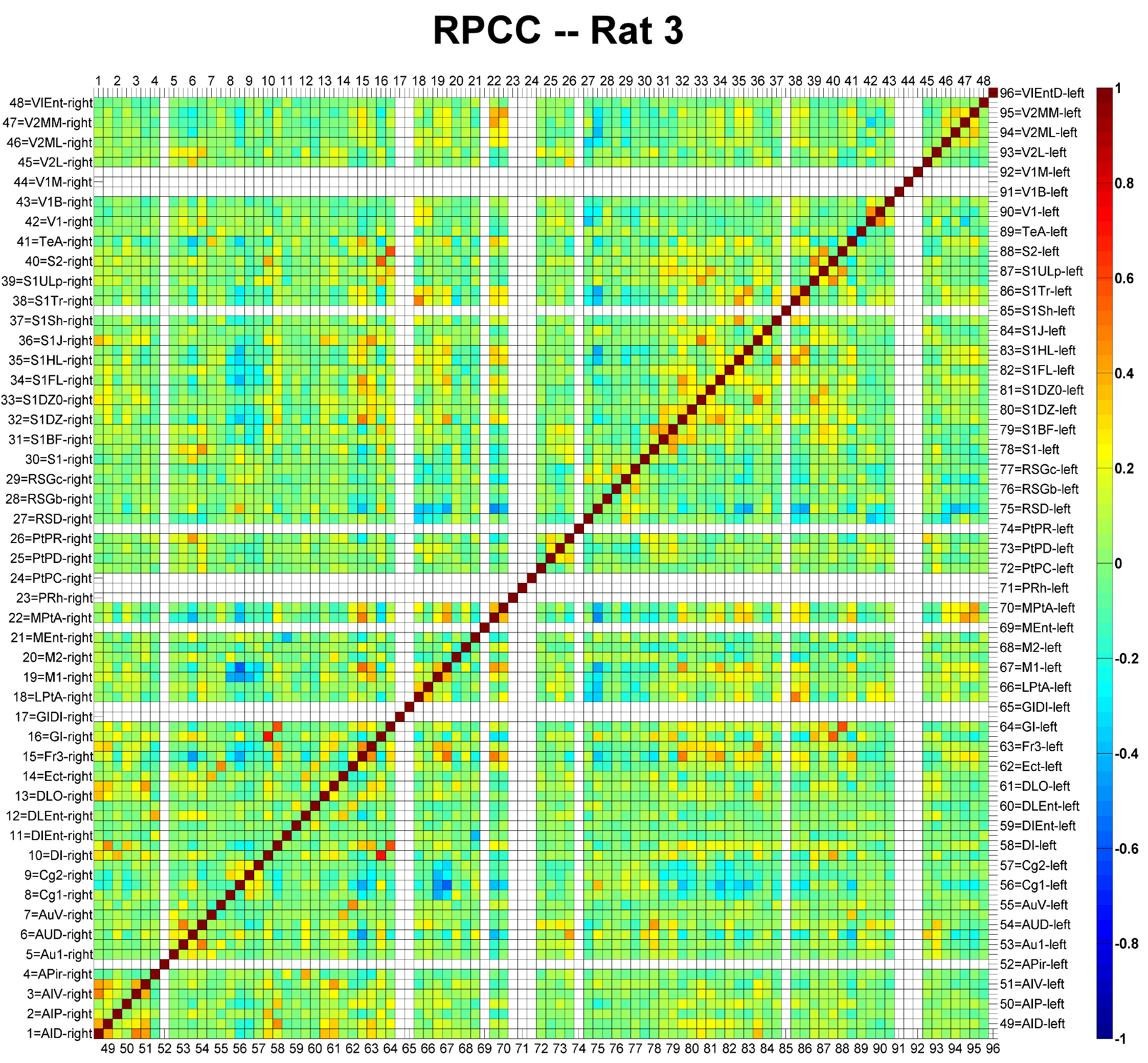

Supplement: S2 Fig — Text same as in S 1. (TIF) [file pone.0134352.s002.tif]

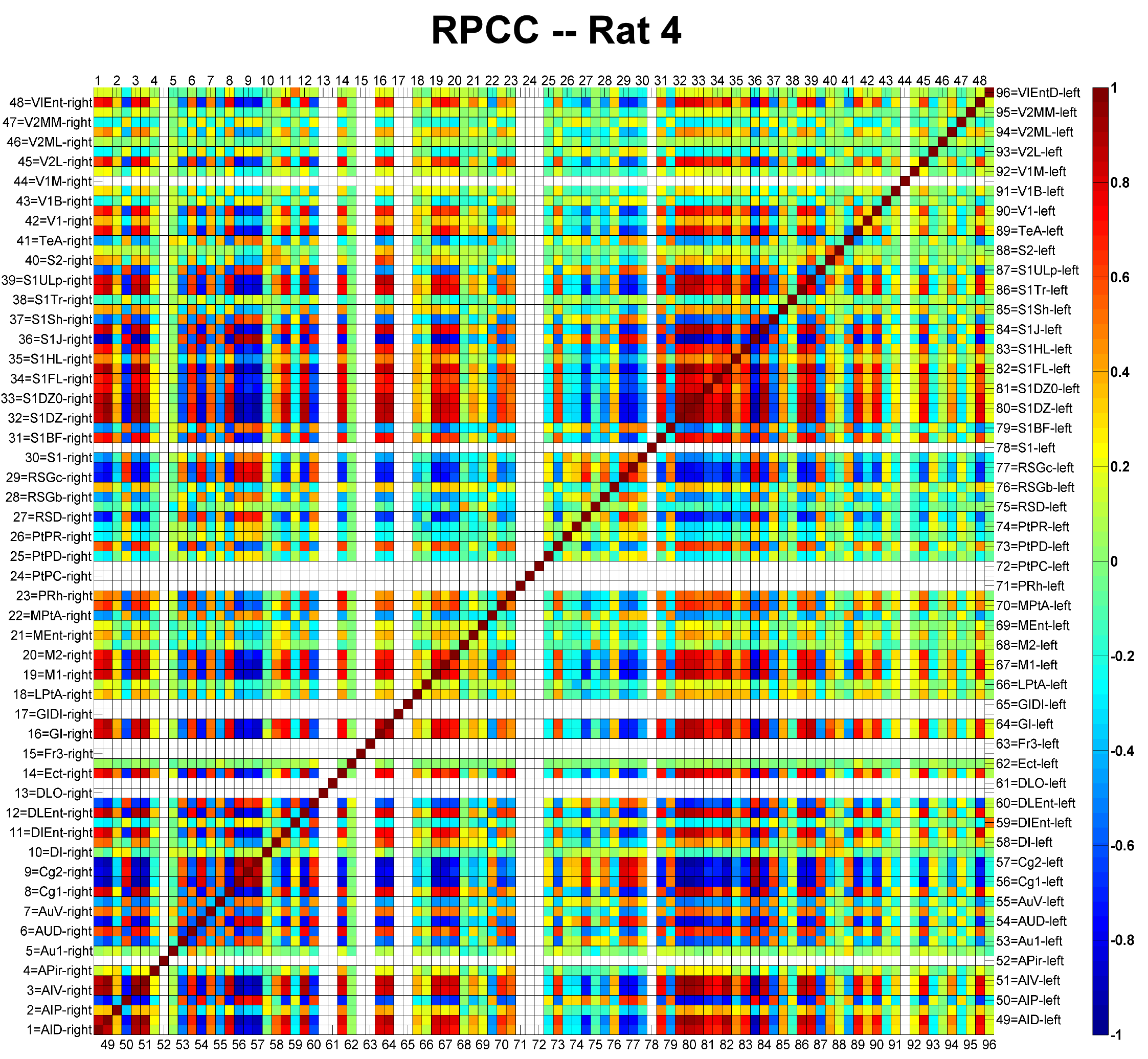

Supplement: S3 Fig — Text same as in S 1. (TIF) [file pone.0134352.s003.tif]

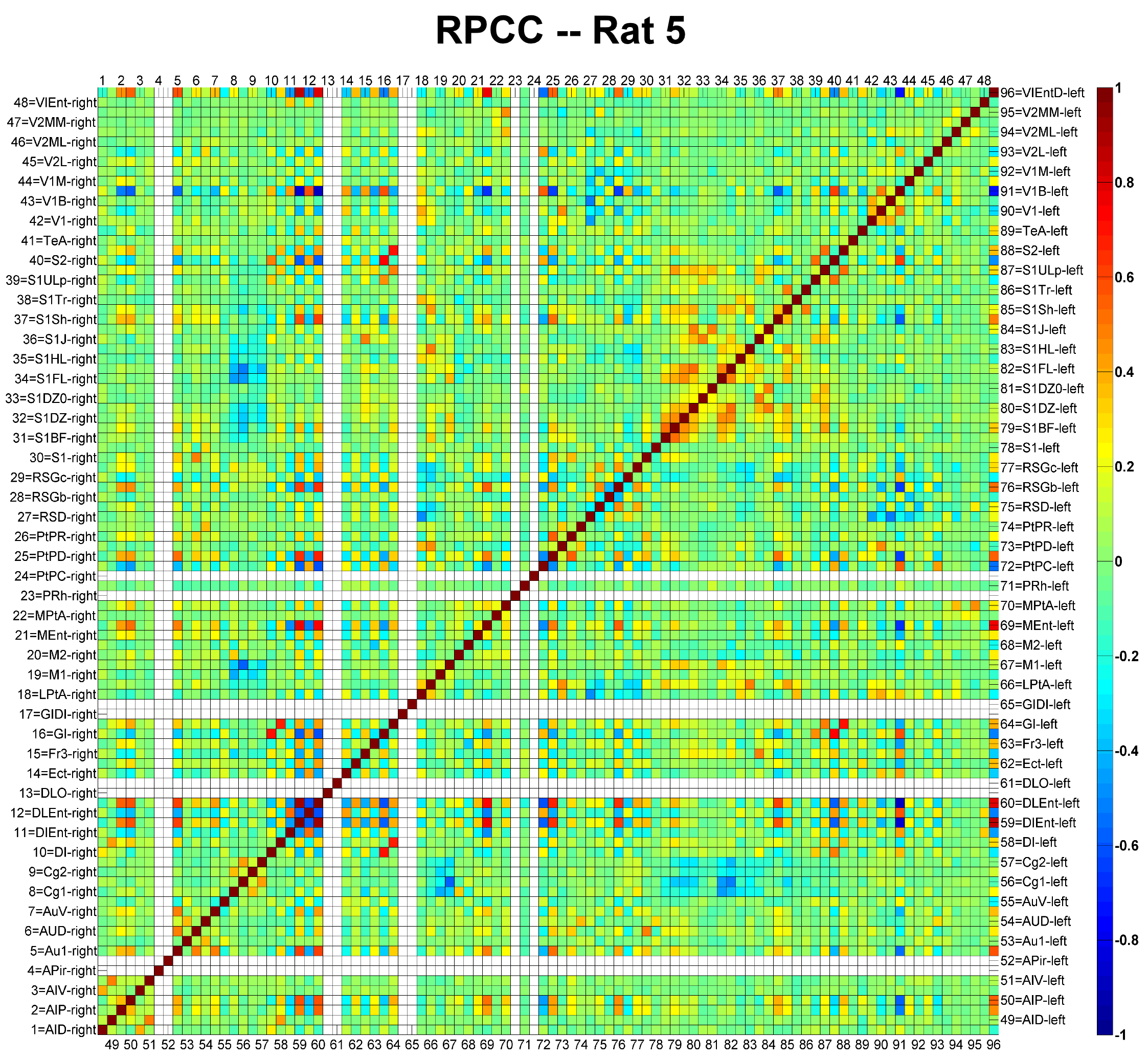

Supplement: S4 Fig — Text same as in S 1. (TIF) [file pone.0134352.s004.tif]

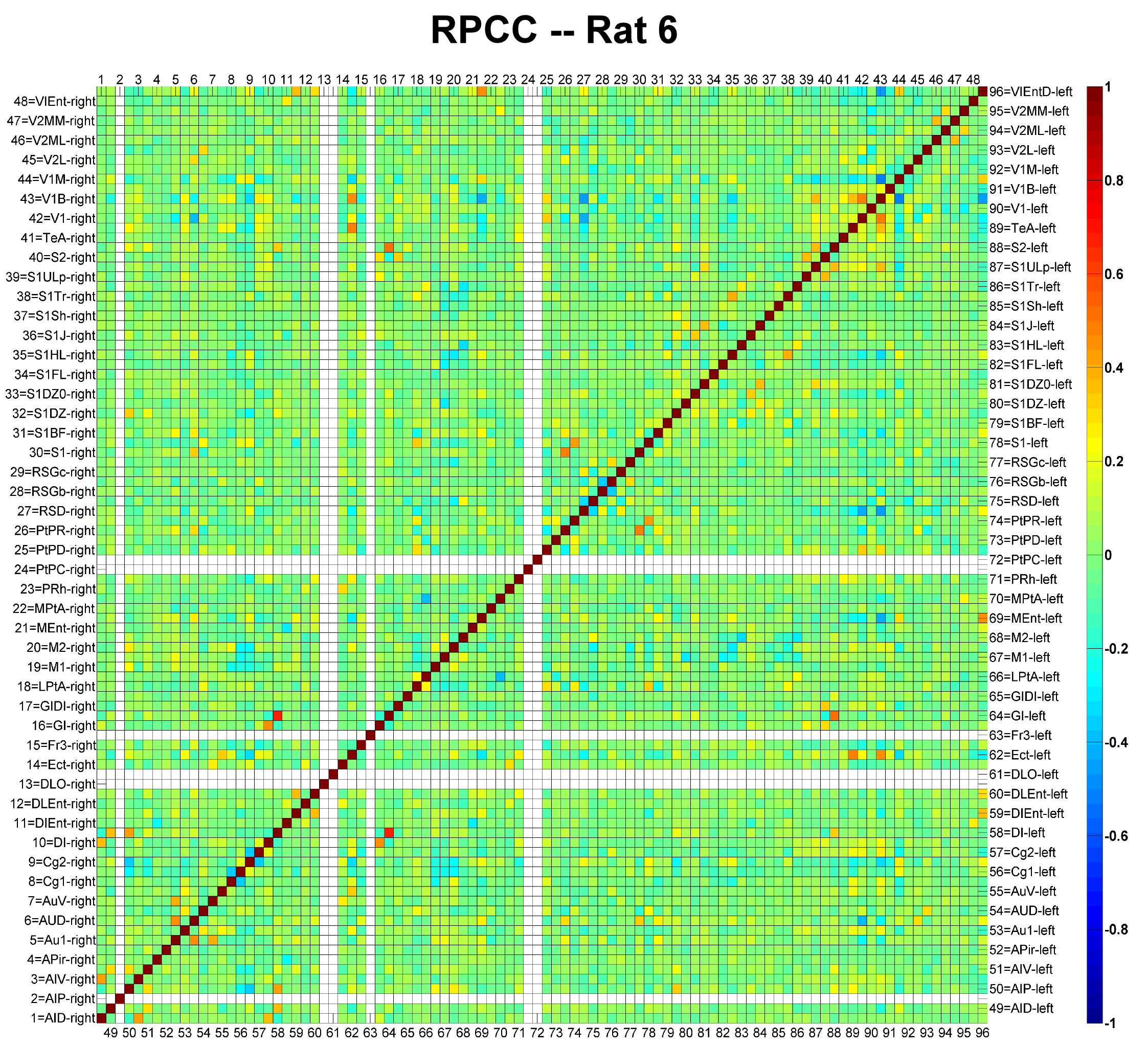

Supplement: S5 Fig — Text same as in S 1. (TIF) [file pone.0134352.s005.tif]
